# Metabolic Engineering of *Saccharomyces cerevisiae* for High-Level Friedelin *via* Genetic Manipulation

**DOI:** 10.3389/fbioe.2022.805429

**Published:** 2022-02-07

**Authors:** Hai-Yun Gao, Huan Zhao, Tian-Yuan Hu, Zhou-Qian Jiang, Meng Xia, Yi-Feng Zhang, Yun Lu, Yuan Liu, Yan Yin, Xiao-Chao Chen, Yun-Feng Luo, Jia-Wei Zhou, Jia-Dian Wang, Jie Gao, Wei Gao, Lu-Qi Huang

**Affiliations:** ^1^ School of Traditional Chinese Medicine, Capital Medical University, Beijing, China; ^2^ School of Pharmacy, College of Medicine, Hangzhou Normal University, Hangzhou, China; ^3^ Beijing Shijitan Hospital, Capital Medical University, Beijing, China; ^4^ School of Chinese Materia Medica, Beijing University of Chinese Medicine, Beijing, China; ^5^ College of Biotechnology and Bioengineering, Zhejiang University of Technology, Hangzhou, China; ^6^ State Key Laboratory Breeding Base of Dao-di Herbs, National Resource Center for Chinese Materia Medica, Chinese Academy of Chinese Medical Sciences, Beijing, China

**Keywords:** friedelin, triterpenes, *Saccharomyces cerevisiae*, genome, CRISPR/Cas9, optimized medium, engineered strain

## Abstract

Friedelin, the most rearranged pentacyclic triterpene, also exhibits remarkable pharmacological and anti-insect activities. In particular, celastrol with friedelin as the skeleton, which is derived from the medicinal plant *Tripterygium wilfordii*, is a promising drug due to its anticancer and antiobesity activities. Although a previous study achieved friedelin production using engineered *Saccharomyces cerevisiae*, strains capable of producing high-level friedelin have not been stably engineered. In this study, a combined strategy was employed with integration of endogenous pathway genes into the genome and knockout of inhibiting genes by CRISPR/Cas9 technology, which successfully engineered multiple strains. After introducing an efficient *Tw*OSC1^T502E^, all strains with genetic integration (*tHMG1*, *ERG1*, *ERG20*, *ERG9*, *POS5*, or *UPC2.1*) showed a 3.0∼6.8-fold increase in friedelin production compared with strain BY4741. Through further double knockout of inhibiting genes, only strains GD1 and GD3 produced higher yields. Moreover, strains GQ1 and GQ3 with quadruple mutants (*bts1*; *rox1*; *ypl062w*; *yjl064w*) displayed similar increases. Finally, the dominant strain GQ1 with *Tw*OSC1^T502E^ was cultured in an optimized medium in shake flasks, and the final yield of friedelin reached 63.91 ± 2.45 mg/L, which was approximately 65-fold higher than that of the wild-type strain BY4741 and 229% higher than that in ordinary SD-His-Ura medium. It was the highest titer for friedelin production to date. Our work provides a good example for triterpenoid production in microbial cell factories and lays a solid foundation for the mining, pathway analysis, and efficient production of valuable triterpenoids with friedelin as the skeleton.

## Introduction

Friedelin is a typical friedelane-type pentacyclic triterpenoid extracted from cork and stem bark of various plants, such as *Quercus suber* (Cristina et al., 2006), *Calophyllum pinetorum* (Bello et al., 2008), and *Drypetes tessmanniana* (Kuete et al., 2010). In recent years, a considerable number of studies have reported that friedelin exhibits remarkable pharmacological activities, such as anti-inflammatory, hypolipidaemic, and antidiabetic activities (Antonisamy et al., 2011; Duraipandiyan et al., 2016; Sunil et al., 2021). In particular, friedelin is the critical precursor of celastrol, which is isolated from the medicinal plant *Tripterygium wilfordii* and represents one of the most promising drugs used in anticancer and antiobesity studies (Zhou et al., 2019). Thus, friedelin and its derivatives provide potential resources for the development of new drugs or dietary supplements. Additionally, friedelin also plays an important role in agriculture. For example, friedelin shows excellent natural anti-insect activity for plant protection (Baskar et al., 2014), and Dong et al. found that friedelin could regulate soil microbial community dynamics as an allelochemical (Dong et al., 2014).

However, the supply of friedelin from plant sources is insufficient, while chemical methods are often complex, include extreme reaction conditions, and produce toxic chemicals; thus, it is urgent to develop an environmentally friendly and cost-effective method of securing the friedelin supply (Moses et al., 2013; Guo et al., 2020). Synthetic biology and metabolic engineering have provided promising and green approaches to reconstruct microorganisms to yield these natural high-value products. Sharing the common upstream biosynthetic pathway with plant triterpenes, *Saccharomyces cerevisiae* could provide the essential precursor 2,3-oxidosqualene, which is cyclized into diverse triterpene skeletons by oxidosqualene cyclases (OSCs) (Thimmappa et al., 2014; Rahimi et al., 2019). In *S. cerevisiae*, the compatibility with most membrane proteins and feasibility of genetic engineering make it a perfect chassis for the production of numerous triterpenes (Carsanba et al., 2021), such as ginsenosides Rh2 (Wang et al., 2019), *α*-amyrin (Yu et al., 2020), and oleanolic acid (Zhao et al., 2018).

**FIGURE 1:**
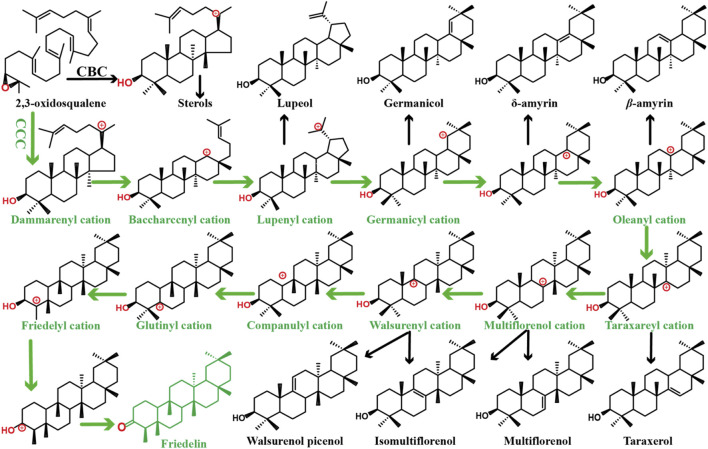
Mechanism for the cyclization of 2,3-oxidosqualene into the most rearranged pentacyclic friedelin and diverse triterpenoid skeletons. The green arrows indicate rearrangement steps including 2,3-oxidosqualene protonation, cyclization, several rearrangements, and deprotonation. The black arrows represent the formation of other triterpenoid skeletons. C–C–C, chair–chair–chair; C–B–C, chair–boat–chair.

Friedelin is the most highly rearranged pentacyclic triterpene in plants, (Han et al., 2019; Zhou et al., 2019; Lu et al., 2021), the cyclization mechanism of which can be seen in Figure 1. In our previous study, friedelin production in engineered yeast was achieved by combining CRISPR/Cas9 technology with plasmid overexpression of genes, and the yield of friedelin was 37.07 mg/L. However, when strain ZH1, stored in the refrigerator at −80°C, was fermented and tested under the same operating conditions, the yield of friedelin was only comparable to that of the wild-type strain BY4741, which was likely caused by the instability of the plasmid (Zhang et al., 1996; Flagfeldt et al., 2009). In the process of strain preservation and activation, the characteristics of the engineered strain were lost due to the loss of plasmids or partial functional fragments, which led to a sharp decline in the production of friedelin. Hansen et al. obtained a haploid strain containing seven deletions, and it showed friedelin production of only 0.5 mg/L (Hansen et al., 2020). Obviously, the low yield of friedelin and instability of the strains limit its further application.

**FIGURE 2:**
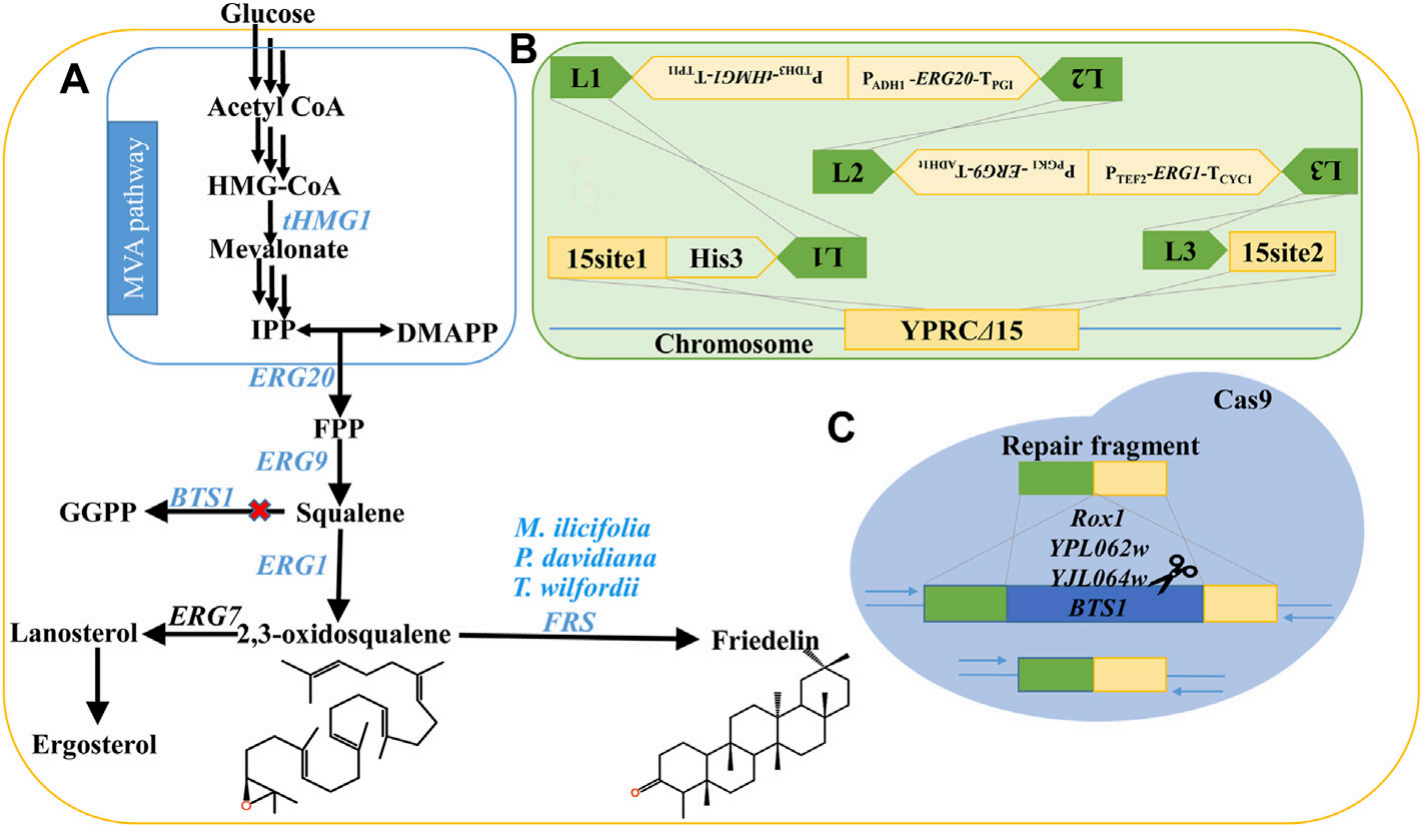
Strategies employed to enhance the production of friedelin in *S. cerevisiae*. **(A)** The biosynthesis pathway of friedelin in *S. cerevisiae*. **(B)** Key genes (*tHMG1*, *ERG20*, *ERG9*, *ERG1*) of the MVA pathway were integrated into the genome of BY4741 to increase the metabolic flux of precursor 2,3-oxidosqualene. **(C)** Knock out genes affecting the MVA pathway by CRISPR/Cas9 technology, including *BTS1*, *ROX1*, *YPL062w* and *YJL064w*. The red X represents blocking. IPP: isopentenyl diphosphate; FPP: farnesyl diphosphate; GGPP: (*E*,*E*,*E*)-geranylgeranyl diphosphate; FRS: friedelin synthase.

In this study, we constructed a stable and high-yielding engineered strain, GQ1, by integrating multiple endogenous genes into yeast chromosomes and regulating genes of competing pathways. These endogenous genes include truncated 3-hydroxyl-3-methylglutaryl-CoA reductase (*tHMG1*), farnesyl diphosphate synthase (*ERG20*), squalene synthase (*ERG9*), and 2,3-oxidosqualene synthase (*ERG1*), which are involved in the mevalonate (MVA) pathway, as well as an uptake control transcriptional regulator (*UPC2.1*) and *POS5* gene (encoded a NADH kinase), which participate in the auxiliary pathway. Overexpression of these genes represented an effective strategy for increasing the metabolic flow of terpenes according to the literature (Dai et al., 2012; Paddon et al., 2013; Dai et al., 2014; Paramasivan and Mutturi, 2017; Li et al., 2020; Sun et al., 2021). At the same time, to further increase the metabolic flux of 2,3-oxidosqualene, the genes *BTS1* (geranylgeranyl diphosphate synthase), *YPL062w*, *YJL064w*, and *ROX1* (repressor of hypoxia) on the competing pathways were knocked out by the CRISPR/Cas9 system (Figure 2). Then, the catalytic efficiency of friedelin synthase (FRS) from different species was evaluated, and *Tw*OSC1^T502E^ from *T. wilfordii* showed the highest efficiency in the production of friedelin. Finally, *Tw*OSC1^T502E^ was transformed into the dominant strain GQ1 and cultured in an optimized medium in shake flasks. The final yield of friedelin was 63.91 ± 2.45 mg/L, which was approximately 65-fold higher than that of the wild-type strain BY4741 and 229% higher than that in ordinary SD-His-Ura medium. To the best of our knowledge, the 63.91 ± 2.45 mg/L yield of friedelin produced in a shake flask represented the highest titer to date. Our work sets a good example for triterpenoid production in microbial cell factories and lays a solid foundation for the mining of valuable triterpenoids with friedelin as the precursor skeleton and the analysis of biosynthesis pathways and efficient synthesis.

## Materials and Methods

### Strains and Medium

The initial strain used in this study was BY4741 (*MATa his3Δ1 leu2Δ0 met15Δ0 ura3Δ0*), which was cultivated in YPD (1% yeast extract (OXOID, England), 2% peptone (OXOID), and 2% glucose) medium. Recombinant yeast strains were selected on synthetic dropout (SD) medium (Fun-Genome Company, China) at 30°C. *E. coli* Trans1-T1 (TransGen Biotech, Beijing, China) was used for plasmid construction, maintenance and amplification, and the bacteria were grown at 37°C in Luria-Bertani (LB) medium (5 g/L yeast extract, 10 g/L tryptone, and 10 g/L NaCl; 20 g/L agar was added for solid medium) with 100 μg/ml ampicillin or 50 μg/ml kanamycin if necessary.

### Construction of Recombinant Strains

#### Integrating Multiple Endogenous Genes Into Yeast Chromosomes

To integrate multiple transcription units simultaneously, a modularized two-step (M2S) technique was performed (Li et al., 2016), and the recombinant strain GH1 was taken as an example. The plasmids used for gene overexpression were stored in our laboratory, and detailed plasmid information is shown in Supplementary Table S1.

The first step is to clone the promoter sequence, gene, and terminator sequence. Overexpressed gene sequences (*Thmg1*, *ERG20*, *ERG9*, *ERG1*, and *POS5*) were downloaded from the Saccharomyces Genome Database (SGD) (yeastgenome.org). The gene sequence of *UPC2.1* was based on Hu. et al. (Hu et al., 2020) and amplified from the *UPC2.1*-Blunt zero plasmid. DNA sequence information of genes is listed in Supplementary Table S5. The genome of *S. cerevisiae* strain BY4741 was extracted as a template to amplify the open reading frame (ORF) of overexpressed genes according to the instructions of the yeast genome extraction kit (Tiagen Biochemical Technology (Beijing) Co., LTD.). According to the sequence information of genes (Ptdh3, Padh1, TPI1t, PGIt, *Thmg1*, and *ERG20*), primers with a Type IIS restriction endonuclease *Bsa*I recognition site (GGTCTCNNNNN) were designed. The promoter Ptdh3-Padh1, terminator TPI1t-PGIt, and genes *Thmg1* and *ERG20* were cloned by PCR with Phusion® High-fidelity DNA Polymerase (New England Biolab, United States) using plasmids P1, T1, and BY4741 as templates. PCR products were recovered by a Gene JET Gel Extraction Kit (TransGen Biotech, Beijing, China). The Golden Gate reaction was performed on the purified DNA fragments with T4 DNA ligase under the following conditions: 25 cycles of 37°C for 3 min and 16°C for 4 min, followed by 50°C for 5 min and 80°C for 5 min, and then held at 4°C. The reaction products were transformed into *E. coli Trans*-T1 competent cells and cultured at 37°C, and then assembled plasmids were sequenced by Tianyibiotech (Beijing, China). The correct plasmid was stored, and overexpression module I was constructed. The results are shown in Supplementary Figure S1. Other overexpression modules were also constructed according to this method. All primers are listed in Supplementary Table S2.

The second step is to construct homologous head and tail arms for integration. Primers were designed based on the sequence information for L1, L4, pESC-His and the *S. cerevisiae* chromosome XVI-15 site (15 site-1: 15 site-1-F/R; His: His (15 site-1)-F/R; L1: L1 (15 site-1)-F/R; L4: L4-15 site2--F/R; 15 site2: 15 site2-F/R). The 15 site1, His, L1, L4, and 15 site2 fragments were cloned by PCR with Phusion® High-fidelity DNA Polymerase (New England Biolab, United States) using plasmids T1 and T3 and genomic DNA as templates. After the PCR products were recovered, overlap PCR was performed to construct head and tail homologous arms, and the results are shown in Supplementary Figure S1.

Finally, these overexpression modules were integrated into the yeast genome in different combinations. For genomic integration, transcription unit modules, selective markers, and integrated homologous arm modules were transformed into *S. cerevisiae* BY4741 cells *via* electroporation. Competent cells were prepared according to the following protocol: single colonies were inoculated in 4 ml liquid YPD to OD600 = 0.6–1.0 and collected *via* centrifugation at 8,000 × g for 1 min. The obtained cell pellet was washed twice using 1 ml precooled sterile water and then incubated in 4 ml transformation reagent (10 mM LiAc, 10 mM DTT, 0.6 M sorbitol, 10 mM pH 7.5 Tris–HCl) for 20 min at 25°C. Conditioned cells were collected by centrifugation, washed twice using 1 ml ice-cold 1 M sorbitol buffer, and then resuspended to a final volume of 100 μL in sorbitol buffer. Cells with 500 ng DNA fragments were electroporated at 3 kV, 25 μF, and 200 Ω (Bio–Rad, Hercules, CA), incubated in 1 ml sorbitol buffer for 2 h at 30°C, and then plated on selective media for 2–3 days.

#### Knocking out Genes in Competing Pathways Using CRISPR Cas9 Technology

The gRNA expression vector p426-SNR52p-gRNA.CAN1. Y-SUP4t (ref. no. 43803), the Cas9 expression vector p414-TEF1p-Cas9-CYC1t (43,802), and the general vectors pTY-U01 and pTY-U02 used for gene knockout were preserved in the laboratory. Specific gRNAs targeting *BTS1*, *ROX1*, *YPL064w*, and *YJL062w* were designed using an open-source tool http://yeastriction.tnw.tudelft.nl, and efficient target sequences were selected (Mans et al., 2015). All gRNA target sequences used in this study are listed in Supplementary Table S3. To obtain the single gRNAs, equal molar ratio solutions of 24 nt oligos F and R were mixed and annealed, resulting in a double-stranded insert with overhangs at both ends. Then, plasmids expressing single gRNAs were constructed by inserting the double-stranded oligos into the *Aar*I recognition site of pTY-U01 using Golden Gate assembly (Engler et al., 2014; Lee et al., 2015). Similar to the construction of the single-gRNA plasmids, *BTS1*-gRNA was efficiently inserted into the plasmid pTY-U02 and resulted in a 2gRNA expression plasmid, and *YJL064w*-gRNA was successfully inserted into the plasmid harbouring *YPL062w*-gRNA (Hu et al., 2020) (Supplementary Figure S3). The details of the reaction conditions are listed, and the primers are shown in Supplementary Table S3.

To obtain strains of specific genotypes, double-stranded DNA (ds-Oligo)-mediated homologous recombination repair is required. Homologous repair fragments of *ROX1*, *BTS1*, *YPL062w*, and *YJL064w* were amplified from the genome of yeast BY4741 (300 bp upstream and downstream of the gene’s ORF) and then spliced by overlapping PCR to obtain the homologous repair fragments of four genes. The primers can be found in Supplementary Table S3. Next, the Cas9 expression vector P414-Leu-TEF1p-Cas9-CYC1t was introduced into GH1, GH2, GH3, and GH4 cells according to the directions of the Frozen-EZ Yeast Transformation II Kit (Zymo Research, Irvine, CA, United States). Four strains with the Cas9 expression plasmid were used for the following manipulations. According to different knockout purposes, a total of 500 ng of the gRNA expression plasmid was mixed with 1 μg of each corresponding homologous repair fragment and electroporated into competent cells containing the Cas9 plasmid. The plasmid information used for gRNA construction is listed in Supplementary Table S4. Electroporation conditions were the same as those mentioned above. After cultivation on synthetic drop-out medium without histidine and leucine at 30°C for 2–3 days, four mutant colonies were selected from each plate, genomic DNA was isolated for use as a template for PCR, and the products were verified by DNA sequencing. The isolated genomic DNA of strain BY4741 was used as a negative control. Then, the gRNA plasmids with *URA*3 labelling were removed with 5-fluoroorotic acid (Laughery et al., 2015), and Cas9 plasmids were removed as described in a previous study (Mans et al., 2015; Hu et al., 2020). The recombinant strains with desired genotypes were used in the following experiments.

### Construction of Friedelin Synthase Expression Plasmids

*Mi*FRS from *Maytenus ilicifolia* (Souza-Moreira et al., 2016) and *Pd*FRS from *Populus davidiana* (Han et al., 2019) (GenBank accession numbers: KX147270.1 and KY931453.1, respectively) were synthesized by Ruibiotech (Beijing, China) and cloned into the cloning sites (BamHI/SalI) of the pESC-Leu plasmid. The sequences of FRSs were downloaded from the National Center for Biotechnology Information (NCBI) database (http://www.ncbi.nlm.nih.gov/gorf/gorf.html). And the codon-optimized plasmid pYES2-*Tw*OSC1^T502E^ from *T. wilfordii* was preserved in the laboratory (GenBank accession number: KY885467.1). These three constructed vectors were transformed into recombinant strains using the Frozen-EZ Yeast Transformation II Kit (Zymo Research, Irvine, CA, United States), and the pYES2 vector and pESC-Leu vector were transformed into yeast as a control. The transformants were screened on the corresponding solid plates and confirmed by colony PCR and DNA sequencing. Plasmid and sequence information is listed in Supplementary Tables S1, S5, respectively.

### Fermentation of Recombinant Strains in Shake Flasks

The positive transformants were then cultured in flasks (100 ml) containing 30 ml of SC-His-Ura and SC-His-Leu medium containing 2% glucose and incubated with shaking at 200 rpm and 30°C for 2 d. Then, the cells were collected and induced in 30 ml of SC-Ura-His medium with 2% galactose in place of glucose and further cultured at 200 rpm and 30°C for 72 h. The dominant strain harbouring pYES2-*Tw*OSC1^T502E^ was cultured in medium containing 5% glucose, 1% yeast extract, 3% peptone, 0.8% KH_2_PO_4_, and 0.6% MgSO_4·_7H_2_O. After culturing at 30°C and 220 rpm for 2 d, the cells were collected and induced in medium containing 5% galactose, 1% yeast extract, 3% peptone, 0.8% KH_2_PO_4_, and 0.6% MgSO_4·_7H_2_O at 30°C and 200 rpm for 72 h. Finally, 10 ml yeast cells were collected and boiled for 15 min with 10 ml 20% KOH and 50% EtOH, the supernatant was extracted with 15 ml hexane, and ultrasound was performed for 30 min, with the process repeated twice. All extracts were combined and then evaporated by rotary evaporation.

### Quantity Analysis

Next, the extracts were dissolved in 1 ml hexane for analysis by gas chromatography and mass spectrometry (GC–MS). For analysis, a 1 μL sample was injected into an Agilent 7000 gas chromatograph in a nonshunt manner using a DB-5 ms capillary column (injector temperature, 305°C) with a DB-5 ms (30 m × 250 μm, film thickness 0.1 μm) capillary column. One microlitre of the concentrated organic phase was then injected at a He flow rate of 1 ml/min with a temperature program of 1 min at 50°C, followed by a gradient from 50 to 260°C at 50°C/min and then to 305°C at 20°C/min, with a 15 min hold. The ion trap temperature was 230°C. The electron energy was 70 eV. Spectra were recorded in the range of 10–550 m/z. Standard chemicals were purchased from YuanYe Biotech (Shanghai, China).

## Results and Discussion

### Recombinant Strains Obtained *via* Co-Expression of Multiple Endogenous Genes

**FIGURE 3:**
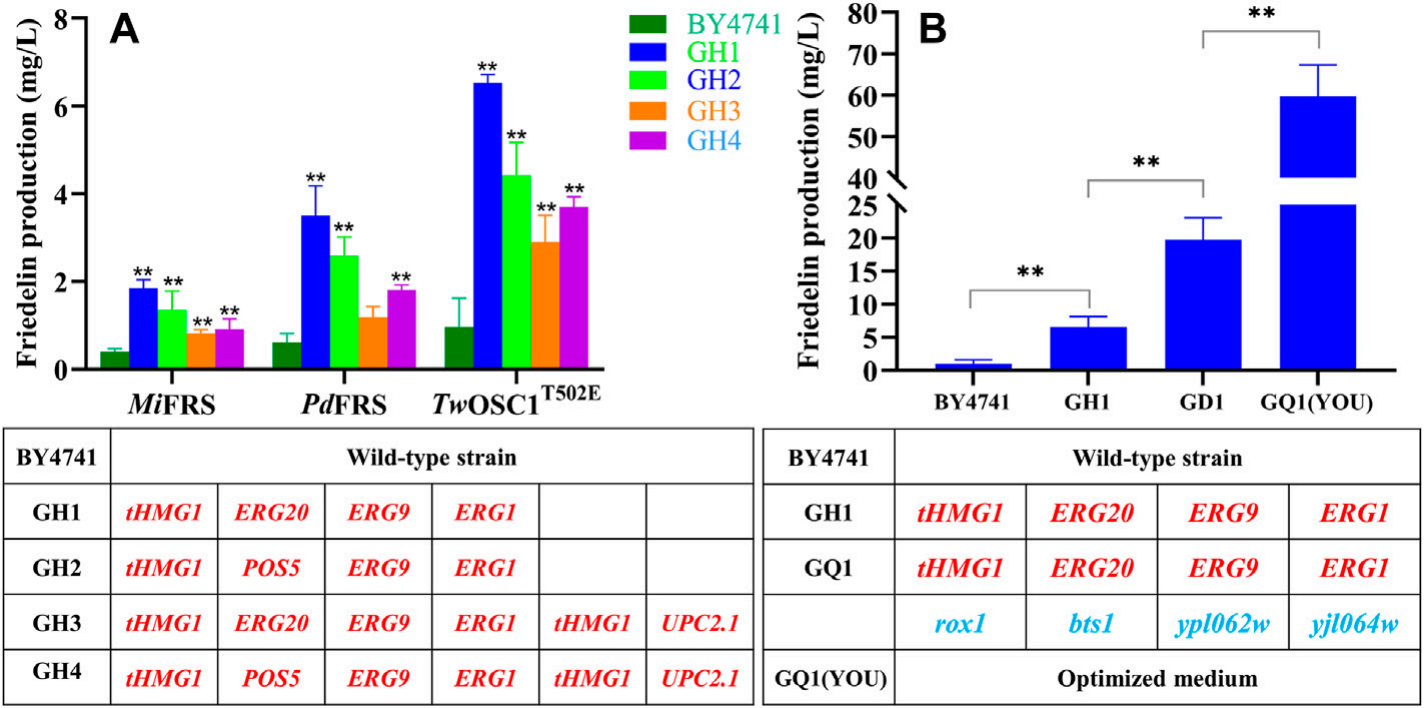
Comparison of the yield of FRSs from different plant sources and the transformation process of the dominant strain GQ1. **(A)** The comparison chart of the yield of friedelin obtained by introducing three FRSs into the recombinant strain GH1, GH2, GH3, GH4, respectively. The asterisk shows a statistical significance between engineered strains and wild-type strain BY4741. **(B)** Diagram of the transformation process of the dominant strain GQ1, (YOU) represents the use of optimized medium for cultivation. The asterisk shows a statistical significance between engineered strains (*p < 0.05, **p < 0.01). In the table below, uppercase red indicates overexpressed genes, and the lowercase blue indicates knocked out genes.

When the functional *Mi*FRS gene in expression plasmid was introduced into the wild-type strain BY4741, the product friedelin reached only 0.39 mg/L (Figure 3A). To raise the yield of friedelin in yeast, a strategy enriching supply of the precursor, 2,3-oxidosqualene, was first considered. In this study, the key genes including *tHMG1*, *ERG20*, *ERG9*, and *ERG1* were integrated into the genome of *S. cerevisiae* BY4741 under the strong promoters P_TDH3_, P_ADH1_, P_PGK1_, and P_TEF2_, leading to the initial recombinant strain GH1. To test the effects of the genomic integrated strains, the *Mi*FRS gene was introduced in plasmid and the friedelin yield was determined by GC-MS. The strain GH1 harbouring *Mi*FRS could produce 4.7-fold friedelin with the control BY4741 (Figure 3A). Some previous cases have proved increased terpene production after these genes’ overexpression, Yu et al. and Wang et al. both overexpressed the *tHMG1*, *ERG20*, *ERG9*, and *ERG1* genes simultaneously in yeast, thereby significantly increasing the production of *α*-amyrin and ginsenoside Rh2 (Wang et al., 2019; Yu et al., 2020), which further demonstrated the feasibility of our research design. *POS5*, which encodes an NADH kinase, can provide more NADPH to serve as electron donor for HMGR and squalene synthase. Various increased yields were achieved with different gene combinations. Thus, an alternative recombinant strain GH2 was generated after replacement of *ERG20* with *POS5*, and yielded 1.35 mg/L of friedelin, which was equivalent to GH1 (Figure 3A). Li et al. verified that the co-expression of *tHMG1* and *POS5* could form a push-pull strategy, thus improving the yield of squalene (Li et al., 2020). In *S. cerevisiae*, overexpression of *tHMG1* has commonly been used to increase the production of many terpenoids, such as artemisinin (Paddon et al., 2013), ginsenosides (Dai et al., 2014), and *β*-carotene (Yan et al., 2012). Multi-copy overexpression of *tHMG1* benefits the production of different terpenoids (Yu et al., 2020), *UPC2.1* enables *S. cerevisiae* to take up external sterols during aerobic cultivation. Therefore, an extra overexpression module with *tHMG1* and *UPC2.1* was constructed in the strains GH1 and GH2. However, the resulting strains GH3 and GH4 showed no more increase compared with strains GH1 and GH2. In brief, the recombinant strains GH1∼GH4 harbouring *Mi*FRS improved the production of friedelin to varying degrees compared with the wild-type strain BY4741, which was GH1>GH2>GH4>GH3 (Figure 3A). The initial recombinant strains GH1 and GH2 had obvious advantages over GH3 and GH4 in friedelin yield improvement through genomic integration. Moreover, to obtain better expression level of the genes, the multiple overexpression cassettes were integrated into the *YPRCΔ15* site of BY4741, because the *YPRCΔ15* site was reported to be one of the integration sites with high *β*-galactosidase activity, and simultaneous integration of multiple genes was achieved at this site (Flagfeldt et al., 2009; Li et al., 2016). The schematic diagrams for overexpression module construction correspond to A, B, C, and D in Supplementary Figure S1. Genotypes of each strain are listed in Table 1.

**TABLE 1:** Strain genotypes and products in this study.

Strain	Host strain	Genotype and (plasmid)	Source
BY4741	*S. cerevisiae*	*MATa his3Δ1 leu2Δ0 met15Δ0 ura3Δ0*	This lab
GH1	BY4741	rDNA: His, P_TDH3_ -*tHMG1*-T_TPI1_-P_ADH1_ -*ERG20*-T_PGI_, P_PGK1_-*ERG9*-T_ADH1_-P_TEF2_-*ERG1*-T_CYC1_	This study
GH2	BY4741	rDNA: His, P_TDH3_ -*tHMG1*-T_TPI1_-P_ADH1_ -*POS5*-T_PGI_, P_PGK1_-*ERG9*-T_ADH1_-P_TEF2_-*ERG1*-T_CYC1_	This study
GH3	BY4741	rDNA: His, P_TDH3_-*tHMG1*-T_TPI1_-P_ADH1_ -*ERG20*-T_PGI_, P_PGK1_ -*ERG9*-T_ADH1_-P_TEF2_-*ERG1*-T_CYC1_,P_FBA_ -*tHMG1*-T_FBA1_-P_HXT7_-*UPC2.1*-T_PDC1_	This study
GH4	BY4741	rDNA: His, P_TDH3_ -*tHMG1*-T_TPI1_-P_ADH1_ -*POS5*-T_PGI_, P_PGK1_ -*ERG9*-T_ADH1_-P_TEF2_-*ERG1*-T_CYC1_, P_FBA_ -*tHMG1*-T_FBA1_-P_HXT7_-*UPC2.1*-T_PDC1_	This study
GD1	GH1	*rox1Δbts1Δ*	This study
GD2	GH2	*rox1Δbts1Δ*	This study
GD3	GH3	*rox1Δbts1Δ*	This study
GD4	GH4	*rox1Δbts1Δ*	This study
GQ1	GH1	*rox1Δbts1Δyjl064wΔ ypl062wΔ*	This study
GQ2	GH2	*rox1Δbts1Δyjl064wΔ ypl062wΔ*	This study
GQ3	GH3	*rox1Δbts1Δyjl064wΔ ypl062wΔ*	This study
GQ4	GH4	*rox1Δbts1Δyjl064wΔ ypl062wΔ*	This study

### Screening Efficient Friedelin Synthase

In the strategy of constructing engineered strains of yeast, the catalytic efficiency of enzymes is another key to realizing high-yield friedelin in addition to a sufficient precursor supply. To date, several FRSs from only four plant species have been functionally characterized (Zhou et al., 2021), among which *Mi*FRS (Souza-Moreira et al., 2016) and *Pd*FRS (Han et al., 2019) were reported to be monofunctional, and others were multifunctional, with friedelin as the major product. Multifunctional *Tw*OSC1^T502E^ proved to significantly increase friedelin production in our previous study, and monofunctional *Mi*FRS and *Pd*FRS were selected and transformed into strains BY4741, GH1, GH2, GH3, and GH4, respectively. The catalytic activity of different FRSs was evaluated in shake flask experiments. Notably, all strains harbouring *Tw*OSC1^T502E^ displayed a higher yield of friedelin than those with *Mi*FRS or *Pd*FRS (Figure 3A **)**. Hence, *Tw*OSC1^T502E^ was finally confirmed as the most efficient FRS and introduced into the recombinant strains for yield evaluation.

Interestingly, the GC–MS results showed that in the extractions of yeast expressing *Pd*FRS and *Mi*FRS, not only friedelin but also *β*-amyrin and *α*-amyrin were detected (Supplementary Figure S2). From the perspective of the complex cyclization mechanism of friedelin (Figure 1 **)**, the production of by-products *β*-amyrin and *α*-amyrin is also reasonable. It was possible that in the functional identification of these two FRS experiments, the amount of 2,3-oxidoxysqualene provided by the chassis strain they used was insufficient, resulting in the by-products not being detected.

### Improving Engineered Strains Through Knockout of Inhibiting Genes

Next, to further improve friedelin production, we downregulated the metabolic flux towards its competing pathways. *BTS1* is the first enzyme in the branching pathway for diterpenoid biosynthesis that catalyses the condensation of farnesyl diphosphate (FPP) and isopentenyl diphosphate (IPP) to (*E*,*E*,*E*)-geranylgeranyl diphosphate (GGPP), which would decrease the 2,3-oxidosqualene supply for triterpene production due to its high consumption of FPP and IPP (Jiang et al., 1995; Tokuhiro et al., 2009). *ROX1* is a transcriptional regulator that represses many genes involved in the MVA pathway and ergosterol synthesis, and *ROX1* deletion has been proven to increase the expression of multiple *ERG* genes, such as *ERG9* and *ERG1* (Henry et al., 2002; Kwast et al., 2002; Jorda and Puig, 2020). Here, two gRNA expression plasmids were constructed to target *BTS1* and *ROX1* (shown in Supplementary Figure S3) and transformed into strains GH1∼GH4, resulting in the double-locus knockout strains GD1∼GD4. Moreover, some studies have reported that simultaneous deletion of genes *YPL062w* and *YJL064w* would increase metabolic flow in the MVA pathway, thus increasing the yield of terpenoids (Giaever et al., 2002; Ozaydin et al., 2013; Chen et al., 2016; Zhou et al., 2019; Hu et al., 2020). Therefore, based on GD1∼GD4, *YPL062w*, and *YJL064w* were then knocked out using the same method as above. Finally, the genomically modified strains GQ1∼GQ4 were obtained with the CRISPR/Cas9 approach.

### Evaluation of the Recombinant Strains

After successful genome editing of multiple genes that would influence friedelin production, the yield of friedelin from the wild-type strain and 12 recombinant strains were evaluated through fermentation in shake flasks. With the introduction of efficient *Tw*OSC1^T502E^, the product friedelin was detected by GC–MS. Statistically significant differences were analysed using the independent samples *t*-test by SPSS software. As shown in Figure 3A, through genetic manipulation, the overexpression strains presented similarly increased production of friedelin when harbouring different FRSs. This result confirmed that the overexpression modules containing *tHMG1*, *ERG20*, *ERG9*, and *ERG1* were critical for high production of friedelin in *S. cerevisiae*. The highest yield was 6.53 ± 0.13 mg/L in the GH1 strain, which was approximately 7-fold higher than that in the wild-type strain BY4741. Many studies have shown that introducing *UPC2.1* into engineered yeast can increase the yield of terpenes or ergosterol (Ro et al., 2006; Dai et al., 2012; Paddon et al., 2013; Paramasivan and Mutturi, 2017; Sun et al., 2021). However, after integration of *UPC2.1* and another copy of *tHMG1* in the background of GH1 and GH2, the resulting strains GH3 and GH4 harbouring FRSs did not show increased harvest (Figure 3A), which indicated that more overexpression modules were not always better. Similar results about *UPC2.1* integration were reported in some studies, and more overexpression cassettes introduced by multiple genetic manipulations would increase the metabolic burden on the strains (Hu et al., 2020). Hu *et al.* failed to improve the GGPP dephosphorylated derivative (*E*,*E*,*E*)-geranylgeraniol (GGOH) production, when *UPC2.1* was integrated into the *YPL062w* deletion site in the strain BY-HZ13. Ro *et al.* found only a modest effect on amorphadiene production when overexpressed in the EPY208 background, which was not significantly improved (Ro et al., 2006). Another possible explanation was that the precursor flux is being channelled to branching pathways. But the ergosterol levels were measured in our study, and were consistent with friedelin production, which did not support the explanation above. The mechanism of *UPC2.1* were illustrated deeply in future work.

Interestingly, strain GH1 with *Tw*OSC1^T502E^ increased friedelin production by 1.85-fold through integration with *ERG20* compared to strain GH2 with *POS5* overexpression in addition to the three common genes (*tHMG1*, *ERG9* and *ERG1*). This phenomenon suggested that *ERG20* was superior to *POS5* for friedelin yield. As Gao reported in 2018, overexpression of *POS*5 gene in strain GW6 with *ERG20*/NADH-*HMGr*/*PPDS*-*ATR1* expression cassette could enhance NADPH supply and produced protopanaxadiol 3∼4.55 fold higher under different sugar fermentations (Gao et al., 2018). Whether overexpression of *ERG20* and *POS5* exhibit synergistic effect needs further research.

**FIGURE 4:**
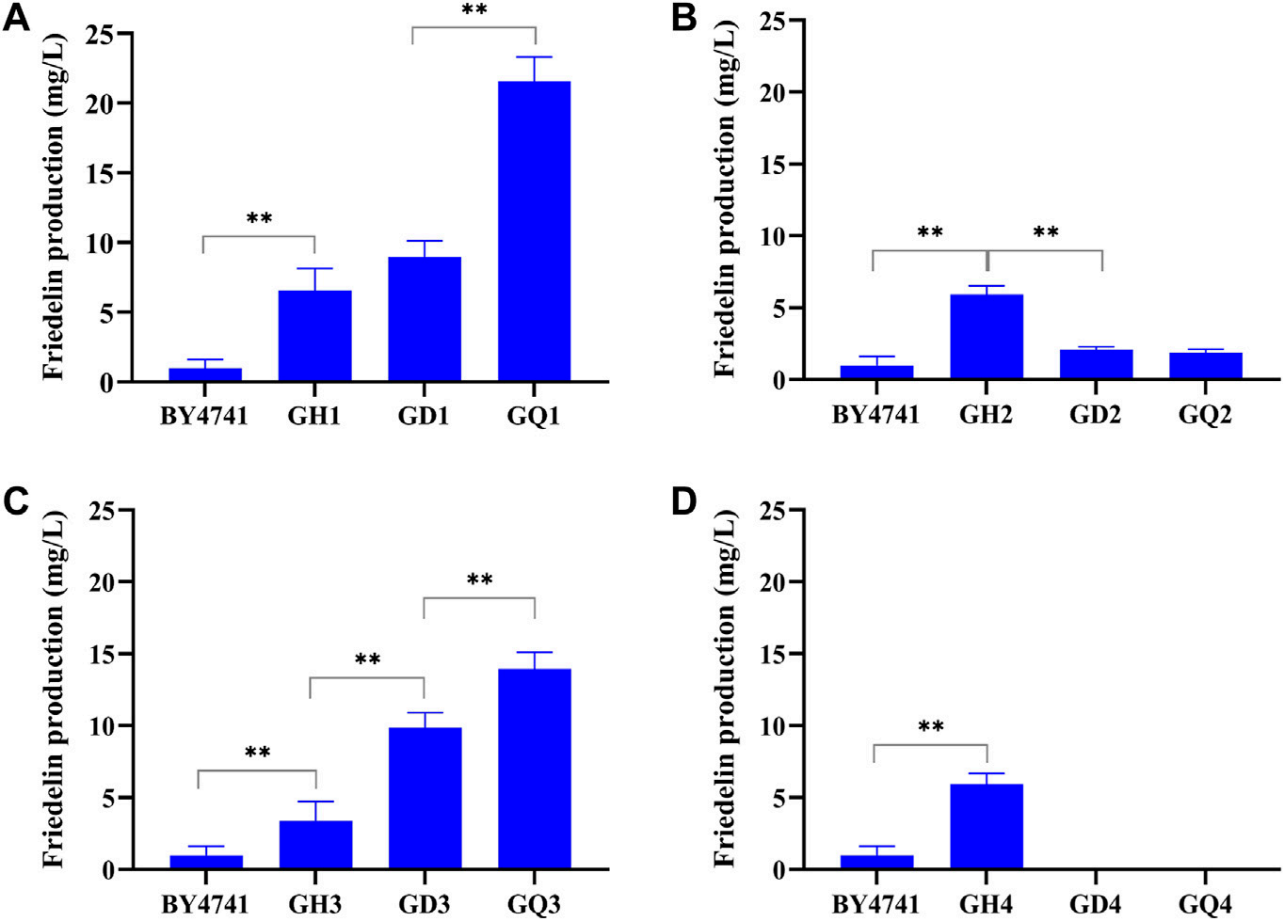
Comparison of the yield of various recombinant strains obtained by overexpressing different gene modules and knocking out different genes. BY4741 was the original strain. GHn stands for different overexpression modules, GDn stands for knockout of two genes on the basis of overexpression, and GQn stands for knockout of 4 genes on the basis of overexpression, *n*=1, 2, 3, 4. **(A)** Friedelin yield of strains GH1, GD1, GQ1. **(B)** Friedelin yield of strains GH2, GD2, GQ2. **(C)** Friedelin yield of strains GH3, GD3, GQ3. **(D)** Friedelin yield of strain GH4. Error bars represented standard deviations of biological quintuples.

Furthermore, when the *ROX1* and *BTS1* genes were knocked out simultaneously, the resulting double-mutant (*rox1*; *bts1*) strains GD1∼GD4 showed different trends of friedelin production (Figure 4). The friedelin yield of strains GD1 and GD3 increased approximately 1.4∼2.9-fold compared with that of strains GH1 (Figure 4A) and GH3 (Figure 4C), while the yield dramatically decreased in strains GD2 (Figure 4B) and GD4 (Figure 4D) when *Tw*OSC1^T502E^ was introduced. Moreover, strain GD4 failed to survive. The growth curve demonstrated that all recombinant strains harbouring *Tw*OSC1^T502E^ had higher OD600 values than BY4741 and had similar growth cycles except strain GH4. The growth remained steady from 96 to 144 h. It is worth mentioning that strain GH4 displayed slower growth than BY4741 and other recombinant strains, and GH4 had no growth after induction (Supplementary Figure S19). The reasonable hypothesis was that the overexpression of cassettes caused a considerable burden to cell growth, and further knock-out led to a loss of activity due to growth pressure. Previous literatures reported some engineered strains exhibited extended cycles (Hu et al., 2020; Yu et al., 2020). This result indicated that the recombinant strains except GH4 had better growth state and the non-extended growth cycle would not consume excessive cost. When *YPL062w* and *YJL064w* were knocked out, the quadruple mutant (*rox1; bts1*; *ypl062w*; *yjl064w*) showed a similar effect, leading to a great increase in friedelin production in the as-constructed strains GQ1 and GQ3, a decrease in GQ2 and even death to GQ4. Notably, among the tested strains, the quadruple mutant GQ1 harbouring *Tw*OSC1^T502E^ had the highest friedelin production level (19.75 ± 2.78 mg/L) in the shake flask experiments, which was approximately 20-fold higher than that of the wild-type strain BY4741 (Figure 4A). These findings suggested that the *POS5* gene in strains GD2 and GD4 may interact negatively with these knockout genes under different genetic backgrounds. Meanwhile, *ERG20* had a synergetic effect with the quadruple mutant genes on friedelin production. In conclusion, the deletion of *ROX1* and *BTS1* had significant effects on friedelin production for strains GH1 and GH3. The recombinant strain GQ1 harbouring *Tw*OSC1^T502E^ was determined to be the dominant strain for a high yield of friedelin.

### Fermentation of Dominant Strain GQ1 With Optimized Medium

From the above study, we screened the dominant strain GQ1 and the highly efficient FRS *Tw*OSC1^T502E^. After transforming *Tw*OSC1^T502E^ into strain GQ1, medium optimization was used to cultivate the dominant strain. Several studies have shown that medium optimization is an effective approach to improving diterpenoid and triterpenoid production (Song et al., 2017; Zhou et al., 2019; Hu et al., 2020). Therefore, we used a medium containing 5% glucose, 1% yeast extract, 3% peptone, 0.8% KH_2_ PO_4_, and 0.6% MgSO_4·_7H_2_O to culture the friedelin high-yield strain GQ1. As shown in Figure 3B, the optimized YPD medium led to a significant increase in friedelin production. The highest yield of friedelin in strain GQ1 under shake-flask conditions was 63.91 ± 2.45 mg/L, which was approximately 65-fold higher than that of the wild-type strain BY4741 and 229% higher than that in ordinary SD-His-Ura medium. Due to the changes in metabolic flux, recombinant strains have different growth conditions and nutritional requirements compared to the original strains. The optimized YPD medium with rich nutrition can precisely meet the high-density growth of engineered bacteria, thereby increasing the yield of friedelin. These results demonstrated that the optimized YPD medium could indeed significantly increase the production of friedelin. To the best of our knowledge, the 63.91 ± 2.45 mg/L yield of friedelin produced in a shake flask was the highest titer to date.

## Conclusion

In this study, a high-yielding friedelin engineered strain was constructed by comprehensive engineering strategies. First, multiple endogenous genes were simultaneously integrated into yeast chromosomes based on the M2S technique, and the yield of friedelin in recombinant strain GH1 was approximately 7-fold higher than that in the wild-type strain BY4741. Then, a transcriptional regulator and three genes that impact the triterpenoid synthesis pathway and the genes of the competitive pathway were knocked out by CRISPR/Cas9 technology, and the yield of friedelin in the quadruple mutant GQ1 (*rox1*; *bts1*; *ypl062w*; *yjl064w*) was approximately 20-fold higher than that in the original strain. Next, the screened high-efficiency FRS *Tw*OSC1^T502E^ was introduced into the dominant strain GQ1 and cultured in optimized medium in shake flasks. The final production of friedelin was 63.91 ± 2.45 mg/L, which was approximately 65-fold higher than that of the wild-type strain and 229% higher than that in ordinary SD-His-Ura medium. To the best of our knowledge, the 63.91 ± 2.45 mg/L yield of friedelin based on shake flask production was the highest titer in heterologous production reported thus far. The engineered yeast strains constructed in this work not only provide new ideas for enhancing friedelin production but can also be used as chassis strains for the mining, pathway analysis, and efficient production of valuable triterpenoids with friedelin as the skeleton.

## Data Availability

The datasets presented in this study can be found in online repositories. The names of the repository/repositories and accession number(s) can be found in the article/Supplementary Material.
