# Supplementary material for: Metabolic Engineering of Saccharomyces cerevisiae for High-Level Friedelin via Genetic Manipulation
Source: Front Bioeng Biotechnol. 2022 Feb 7;10:805429. doi: 10.3389/fbioe.2022.805429 (PMC8859104; doi:10.3389/fbioe.2022.805429)
Supplement: Supplementary file 1 [file DataSheet1.doc]

Supplementary Material

# Metabolic engineering of *Saccharomyces cerevisiae* for high-level friedelin via genetic manipulation

Hai-Yun Gao^1#^, Huan Zhao^1#^, Tian-yuan Hu^2^, Zhou-qian Jiang^1^, Meng Xia^1^, Yi-Feng Zhang^3^, Yun Lu^1^, Yuan Liu^1^, Yan yin^4^, Xiao-chao Chen^1^,Yun-feng Luo^1^, Jia-wei Zhou^5^, Jia-dian Wang^1^, Jie Gao^1^, Wei Gao^1,3*^, Lu-qi Huang^6*^

^1^ School of Traditional Chinese Medicine, Capital Medical University, Beijing 100069, China

^2^ School of Pharmacy, College of Medicine, Hangzhou Normal University, Hangzhou, 311121, China

^3^ Beijing Shijitan Hospital, Capital Medical University, Beijing 100038, China

^4^ School of Chinese Materia Medica, Beijing University of Chinese Medicine, Beijing 102488, China

^5^ College of Biotechnology and Bioengineering, Zhejiang University of Technology, Zhejiang, 310014, China

^6^ State Key Laboratory Breeding Base of Dao-di Herbs, National Resource Center for Chinese Materia Medica, Chinese Academy of Chinese Medical Sciences, Beijing 100700, China

***** Corresponding authors:

Luqi Huang

E-mail: [huangluqi01@126.com](mailto:huangluqi01@126.com)

Wei Gao

E-mail: [weigao@ccmu.edu.cn](mailto:weigao@ccmu.edu.cn)

^#^ Hai-yun Gao and Huan Zhao contributed equally to this work.

**Supplementary Tables**

**Table S1 List of plasmids used in this study.**

| **Plasmid** | **Genotypes** | **Source** |
| --- | --- | --- |
| pMD-19T | Amp+ | Synbio-Tech |
| pUC57-Kan | Kan+ | Synbio-Tech |
| P1(pTDH3-pADH1) | Amp+ promoter vector with promoters TDH3 and ADH1 | Laboratory preservation |
| P2(pPGK1-pTEF2) | Amp+ promoter vector with promoters PGK1 and TEF2 | Laboratory preservation |
| P3(pFBA-pHXT7) | Amp+ promoter vector with promoters FBA and HXT7 | Laboratory preservation |
| T1-(TPI1t-PGIt) | Kan+ terminator vector, contains terminators TPI1 and PGI | Laboratory preservation |
| T2-(ADH1t -tCYC1t) | Kan+ terminator vector with terminators ADH1 and CYC1 | Laboratory preservation |
| T3-(FBA1t -tPDC1t) | Kan+ terminator vector with terminators FBA1 and PDC1 | Laboratory preservation |
| pESC-LEU-*Pd*FRS  pESC-LEU-*Mi*FRS | 2μ; *LEU2; AmpR;* P*_GAL1_*- *Pd*FRS -*T_CYC1_*  2μ; *LEU2; AmpR;* P*_GAL1_*- *Mi*FRS -*T_CYC1_* | Synbio-Tech  Synbio-Tech |
| pYES2-*Tw*OSC1^T502E^ | 2μ; *URA3; AmpR* | Laboratory preservation |
| pESC-HIS | 2μ; *HIS3; AmpR* | Laboratory preservation |

# Table S2 List of primers for genome integration.

| **Primer name** | **Sequence (5′-3′)** |
| --- | --- |
| 15site-1-F | ATAAAGCAGCCGCTACCAAACAGACAAGATTCAGTATG |
| 15site-1-R  HIS3(15site-1)-F | TATAGAGTGTACTAGTTGGTAAAGCCTATAATATTATGT  TTATAGGCTTTACCAACTAGTACACTCTATATTTTTTTATGCCT |
| HIS3(15site-1)-R | AACGGACCGGGG CTACATAAGAACACCTTTGGTGGAG |
| L1(15site-1)-F | AGGTGTTCTTATGTAGCCCCGGTCCGTTTGTTCTATACTTC |
| L1(15site-1)-R | AGGCAATTTTAGAGGGGACTTCTGACTCCCCTGCTTTG |
| 15site-2-F | CAATGGAATTTCGACAATTATCATATTATTC |
| 15site-2-R | GAAATGTAGATTCATTTTGTAGATTCCTATATC |
| L2-15site-2-F | GACAAAGCGCCAAGGAACTGTAATATATAGCTACGC |
| L2-15site-2-R | AATTGTCGAAATTCCATTGTAGTCCGCGAGTTGGATAGC |
| P1-BsaI-F | CTCTGGTCTCACATCTTTGTTTGTTTATGTG |
| P1-BsaI-R | CTCTGGTCTCAATTCTGTATATGAGATAGTTG |
| T1-BsaI-F | CTCGGTCTCAATCGAACAAATCGCTCTTAA |
| T1-BsaI-R | GAATTCGGTCTCAACCTGATTAATATAAT |
| L1-F | CCCCGGTCCGTTTGTTCTATACTTC |
| pTDH3-R | GACGGTAGGTATTGATTGTAATTCTG |
| L1-pADH1-R | GATGGCAAATGGAAAAAGGGTAGTG |
| pADH1-L2-F | ACATGTAGGTGGCGGAGGGGAGAT |
| pADH1-F | GTTTCCTCGTCATTGTTCTCGTTCC |
| L2-R  P2-BsaI-F  P2-BsaI-R  T2-BsaI-F  T2-BsaI-F  2-L2-F:  pPGK1-R:  pTEF2-F:  2-L3-R:  P3-BsaI-F  P3-BsaI-R  T3-BsaI-F  T3-BsaI-R  3-L3-F:  pFBA-R:  pHXT7-F:  3-L4-R:  *tHMG1*-BsaI-F  *tHMG1*-BsaI-R  *ERG20*-BsaI-F  *ERG20*-BsaI-R  *ERG9*-BsaI-F  *ERG9*-BsaI-R  *ERG1*-BsaI-F  *ERG1*-BsaI-R  *tHMG1*-BsaI-F  *tHMG1*-BsaI-R  *UPC2.1*-BsaI-F  *UPC2.1*-BsaI-R  *POS5*-BsaI-F  *POS5*-BsaI-R | TAGTCCGCGAGTTGGATAGCCCGAG  CTCTGGTCTCACATCTGTTTTATATTTGTTGAAA  CTCTGGTCTCAATTCGGTACTAGTGTTTAGTTAAT  CTCGGTCTCAATCGTCATGTAATTAGTTATGTCA  GAATTCGGTCTCAACCTAGTTATAAAAAAAATAAGTG  GACAAAGCGCCAAGGAACTGTAATA  TACATATTTGGTCTTTTCTAATTCG  ACTTGTTTATTCCCTTCAAGGTTTT  GCGGACTTAGTCCGTTTCTCGGCTA  CCAGGTCTCACATC TTTGAATATGTATTACTTGG  CCAGGTCTCAATTC TTTTTGATTAAAATTAAAAA  CCAGGTCTCAATCG GCGATTTAATCTCTAATTAT  CCAGGTCTCAACCT GTTAATTCAAATTAATTGAT  AACGACGGTAGACGCCAACTACGCT  ACATAATACTTCTCTATCAATTTCA  CTATTCTTCATTTGCAGCTATTGTA  AGGTTCCAACTGCTCTTACTGTAGT  CCAGGTCTCAGATGGACCAATTGGTGAAAACTG  CCAGGTCTCAAGGTTTAGGATTTAATGCAGGTGACGGAC  CCAGGTCTCAGAATATGGCTTCAGAAAAAGAAATTA  CCAGGTCTCACGATCTATTTGCTTCTCTTGTAAACTTTG  CCAGGTCTC AGATG GGAAAGCTATTACAATTGG  CCAGGTCTC AAGGT TCACGCTCTGTGTAAAGTGTATATA  CCAGGTCTC AGAAT ATGTCTGCTGTTAACGTTGCACC  CCAGGTCTC ACGAT TTAACCAATCAACTCACCAAACAAA  CCAGGTCTC AGATG GACCAATTGGTGAAAACTG  CCAGGTCTC AAGGT TTAGGATTTAATGCAGGTGACGGAC  CCAGGTCTC AGAAT AGGAGCAACAGTAGTGAAGTAT  CCAGGTCTC ACGAT TCATAACGAAAAATCAGAGAAAT  CCAGGTCTCAGAATATGTTTGTCAGGGTTAAATTGAATAAACCAGTAAACCAGGTCTCACGATTTAATCATTATCAGTCTGTCTCTTGGTCAGCC |

***** The sequences with black underline are recognition site of type IIS restriction enzyme *Bsa*I.

# Table S3 Primers of gRNA oligo and repair oligo.

| **Primers** | **Sequences** |
| --- | --- |
| BTS1_targetRNA FW  BTS1_targetRNA RV  YJL064W_targetRNA FW | GATCTAACAATTCGTCATGTAAAT  AAACATTTACATGACGAATTGTTA  GATCCACTTGTAGTATATCTAACT |
| YJL064W_targetRNA RV | AAACAGTTAGATATACTACAAGTG |
| YPL062W_targetRNA FW | GATCTCGCTTTAACTTTACCGTTT |
| ROX1_repair oligo F  ROX1_repair oligo R  BTS1_repair oligo F  BTS1_repair oligo R  YJL064w_ repair oligo F  YJL064w_ repair oligo R  YPL062w_ repair oligo F  YPL062w_ repair oligo R  TY-F (Clone fragments with gRNA)  TY-R (Clone fragments with gRNA) | CATTATTCCAGAAAATACTAATACT  TTAGTTAAAGGGAATATAGTATAAT  GACAAGCCAATACTGAAAGAAAGAA  TCGGAAAAATTAGGTCATATACTCC  TCTTCGTCGTCGTCTTCGTC  GTAGGGCTAACGACTCAGCG  CGTACTCACAACTTTCCGCG  GGTGGCAGAAGAGACCTCAC  GGGAAACGCCTGGTATCTTTA  TGTGCTGCAAGGCGATTAAG |

#

# Table S4 List of gRNA plasmids information used in this study.

| **Name** | **Description** | **Source** | |
| --- | --- | --- | --- |
| p414 | TRP1-TEF1p-Cas9-CYC1t | | Laboratory preservation |
| P426 | URA3-SNR52p-gRNA.CAN1.Y-SUP4t | | Laboratory preservation |
| pTY-U01 | p426::*Aar*I | | Laboratory preservation |
| pTY-U02 | p426::*rox*1gRNA+*Aar*I | | Laboratory preservation |
| pgRNA-01 | p426:: *bts*1 | | This study |
| pgRNA-02 | p426:: *rox*1 | | This study |
| pgRNA-03 | p426:: *yjl064w* | | This study |
| pgRNA-04 | p426:: *ypl062w* | | This study |
| 2gRNA-05 | p426:: *bts1*+ *rox1* | | This study |

**Table S5** DNA sequences of genes used in this study.

| **Genes** | **DNA sequences** |
| --- | --- |
| *tHMG1* | GACCAATTGGTGAAAACTGAAGTCACCAAGAAGTCTTTTACTGCTCCTGTACAAAAGGCTTCTACACCAGTTTTAACCAATAAAACAGTCATTTCTGGATCGAAAGTCAAAAGTTTATCATCTGCGCAATCGAGCTCATCAGGACCTTCATCATCTAGTGAGGAAGATGATTCCCGCGATATTGAAAGCTTGGATAAGAAAATACGTCCTTTAGAAGAATTAGAAGCATTATTAAGTAGTGGAAATACAAAACAATTGAAGAACAAAGAGGTCGCTGCCTTGGTTATTCACGGTAAGTTACCTTTGTACGCTTTGGAGAAAAAATTAGGTGATACTACGAGAGCGGTTGCGGTACGTAGGAAGGCTCTTTCAATTTTGGCAGAAGCTCCTGTATTAGCATCTGATCGTTTACCATATAAAAATTATGACTACGACCGCGTATTTGGCGCTTGTTGTGAAAATGTTATAGGTTACATGCCTTTGCCCGTTGGTGTTATAGGCCCCTTGGTTATCGATGGTACATCTTATCATATACCAATGGCAACTACAGAGGGTTGTTTGGTAGCTTCTGCCATGCGTGGCTGTAAGGCAATCAATGCTGGCGGTGGTGCAACAACTGTTTTAACTAAGGATGGTATGACAAGAGGCCCAGTAGTCCGTTTCCCAACTTTGAAAAGATCTGGTGCCTGTAAGATATGGTTAGACTCAGAAGAGGGACAAAACGCAATTAAAAAAGCTTTTAACTCTACATCAAGATTTGCACGTCTGCAACATATTCAAACTTGTCTAGCAGGAGATTTACTCTTCATGAGATTTAGAACAACTACTGGTGACGCAATGGGTATGAATATGATTTCTAAAGGTGTCGAATACTCATTAAAGCAAATGGTAGAAGAGTATGGCTGGGAAGATATGGAGGTTGTCTCCGTTTCTGGTAACTACTGTACCGACAAAAAACCAGCTGCCATCAACTGGATCGAAGGTCGTGGTAAGAGTGTCGTCGCAGAAGCTACTATTCCTGGTGATGTTGTCAGAAAAGTGTTAAAAAGTGATGTTTCCGCATTGGTTGAGTTGAACATTGCTAAGAATTTGGTTGGATCTGCAATGGCTGGGTCTGTTGGTGGATTTAACGCACATGCAGCTAATTTAGTGACAGCTGTTTTCTTGGCATTAGGACAAGATCCTGCACAAAATGTTGAAAGTTCCAACTGTATAACATTGATGAAAGAAGTGGACGGTGATTTGAGAATTTCCGTATCCATGCCATCCATCGAAGTAGGTACCATCGGTGGTGGTACTGTTCTAGAACCACAAGGTGCCATGTTGGACTTATTAGGTGTAAGAGGCCCGCATGCTACCGCTCCTGGTACCAACGCACGTCAATTAGCAAGAATAGTTGCCTGTGCCGTCTTGGCAGGTGAATTATCCTTATGTGCTGCCCTAGCAGCCGGCCATTTGGTTCAAAGTCATATGACCCACAACAGGAAACCTGCTGAACCAACAAAACCTAACAATTTGGACGCCACTGATATAAATCGTTTGAAAGATGGGTCCGTCACCTGCATTAAATCCTAA |
| *ERG20* | ATGGCTTCAGAAAAAGAAATTAGGAGAGAGAGATTCTTGAACGTTTTCCCTAAATTAGTAGAGGAATTGAACGCATCGCTTTTGGCTTACGGTATGCCTAAGGAAGCATGTGACTGGTATGCCCACTCATTGAACTACAACACTCCAGGCGGTAAGCTAAATAGAGGTTTGTCCGTTGTGGACACGTATGCTATTCTCTCCAACAAGACCGTTGAACAATTGGGGCAAGAAGAATACGAAAAGGTTGCCATTCTAGGTTGGTGCATTGAGTTGTTGCAGGCTTACTTCTTGGTCGCCGATGATATGATGGACAAGTCCATTACCAGAAGAGGCCAACCATGTTGGTACAAGGTTCCTGAAGTTGGGGAAATTGCCATCAATGACGCATTCATGTTAGAGGCTGCTATCTACAAGCTTTTGAAATCTCACTTCAGAAACGAAAAATACTACATAGATATCACCGAATTGTTCCATGAGGTCACCTTCCAAACCGAATTGGGCCAATTGATGGACTTAATCACTGCACCTGAAGACAAAGTCGACTTGAGTAAGTTCTCCCTAAAGAAGCACTCCTTCATAGTTACTTTCAAGACTGCTTACTATTCTTTCTACTTGCCTGTCGCATTGGCCATGTACGTTGCCGGTATCACGGATGAAAAGGATTTGAAACAAGCCAGAGATGTCTTGATTCCATTGGGTGAATACTTCCAAATTCAAGATGACTACTTAGACTGCTTCGGTACCCCAGAACAGATCGGTAAGATCGGTACAGATATCCAAGATAACAAATGTTCTTGGGTAATCAACAAGGCATTGGAACTTGCTTCCGCAGAACAAAGAAAGACTTTAGACGAAAATTACGGTAAGAAGGACTCAGTCGCAGAAGCCAAATGCAAAAAGATTTTCAATGACTTGAAAATTGAACAGCTATACCACGAATATGAAGAGTCTATTGCCAAGGATTTGAAGGCCAAAATTTCTCAGGTCGATGAGTCTCGTGGCTTCAAAGCTGATGTCTTAACTGCGTTCTTGAACAAAGTTTACAAGAGAAGCAAATAG |
| *ERG9* | ATGGGAAAGCTATTACAATTGGCATTGCATCCGGTCGAGATGAAGGCAGCTTTGAAGCTGAAGTTTTGCAGAACACCGCTATTCTCCATCTATGATCAGTCCACGTCTCCATATCTCTTGCACTGTTTCGAACTGTTGAACTTGACCTCCAGATCGTTTGCTGCTGTGATCAGAGAGCTGCATCCAGAATTGAGAAACTGTGTTACTCTCTTTTATTTGATTTTAAGGGCTTTGGATACCATCGAAGACGATATGTCCATCGAACACGATTTGAAAATTGACTTGTTGCGTCACTTCCACGAGAAATTGTTGTTAACTAAATGGAGTTTCGACGGAAATGCCCCCGATGTGAAGGACAGAGCCGTTTTGACAGATTTCGAATCGATTCTTATTGAATTCCACAAATTGAAACCAGAATATCAAGAAGTCATCAAGGAGATCACCGAGAAAATGGGTAATGGTATGGCCGACTACATCTTAGATGAAAATTACAACTTGAATGGGTTGCAAACCGTCCACGACTACGACGTGTACTGTCACTACGTAGCTGGTTTGGTCGGTGATGGTTTGACCCGTTTGATTGTCATTGCCAAGTTTGCCAACGAATCTTTGTATTCTAATGAGCAATTGTATGAAAGCATGGGTCTTTTCCTACAAAAAACCAACATCATCAGAGATTACAATGAAGATTTGGTCGATGGTAGATCCTTCTGGCCCAAGGAAATCTGGTCACAATACGCTCCTCAGTTGAAGGACTTCATGAAACCTGAAAACGAACAACTGGGGTTGGACTGTATAAACCACCTCGTCTTAAACGCATTGAGTCATGTTATCGATGTGTTGACTTATTTGGCCGGTATCCACGAGCAATCCACTTTCCAATTTTGTGCCATTCCCCAAGTTATGGCCATTGCAACCTTGGCTTTGGTATTCAACAACCGTGAAGTGCTACATGGCAATGTAAAGATTCGTAAGGGTACTACCTGCTATTTAATTTTGAAATCAAGGACTTTGCGTGGCTGTGTCGAGATTTTTGACTATTACTTACGTGATATCAAATCTAAATTGGCTGTGCAAGATCCAAATTTCTTAAAATTGAACATTCAAATCTCCAAGATCGAACAGTTTATGGAAGAAATGTACCAGGATAAATTACCTCCTAACGTGAAGCCAAATGAAACTCCAATTTTCTTGAAAGTTAAAGAAAGATCCAGATACGATGATGAATTGGTTCCAACCCAACAAGAAGAAGAGTACAAGTTCAATATGGTTTTATCTATCATCTTGTCCGTTCTTCTTGGGTTTTATTATATATACACTTTACACAGAGCGTGA |
| *ERG1* | ATGTCTGCTGTTAACGTTGCACCTGAATTGATTAATGCCGACAACACAATTACCTACGATGCGATTGTCATCGGTGCTGGTGTTATCGGTCCATGTGTTGCTACTGGTCTAGCAAGAAAGGGTAAGAAAGTTCTTATCGTAGAACGTGACTGGGCTATGCCTGATAGAATTGTTGGTGAATTGATGCAACCAGGTGGTGTTAGAGCATTGAGAAGTCTGGGTATGATTCAATCTATCAACAACATCGAAGCATATCCTGTTACCGGTTATACCGTCTTTTTCAACGGCGAACAAGTTGATATTCCATACCCTTACAAGGCCGATATCCCTAAAGTTGAAAAATTGAAGGACTTGGTCAAAGATGGTAATGACAAGGTCTTGGAAGACAGCACTATTCACATCAAGGATTACGAAGATGATGAAAGAGAAAGGGGTGTTGCTTTTGTTCATGGTAGATTCTTGAACAACTTGAGAAACATTACTGCTCAAGAGCCAAATGTTACTAGAGTGCAAGGTAACTGTATTGAGATATTGAAGGATGAAAAGAATGAGGTTGTTGGTGCCAAGGTTGACATTGATGGCCGTGGCAAGGTGGAATTCAAAGCCCACTTGACATTTATCTGTGACGGTATCTTTTCACGTTTCAGAAAGGAATTGCACCCAGACCATGTTCCAACTGTCGGTTCTTCGTTTGTCGGTATGTCTTTGTTCAATGCTAAGAATCCTGCTCCTATGCACGGTCACGTTATTCTTGGTAGTGATCATATGCCAATCTTGGTTTACCAAATCAGTCCAGAAGAAACAAGAATCCTTTGTGCTTACAACTCTCCAAAGGTCCCAGCTGATATCAAGAGTTGGATGATTAAGGATGTCCAACCTTTCATTCCAAAGAGTCTACGTCCTTCATTTGATGAAGCCGTCAGCCAAGGTAAATTTAGAGCTATGCCAAACTCCTACTTGCCAGCTAGACAAAACGACGTCACTGGTATGTGTGTTATCGGTGACGCTCTAAATATGAGACATCCATTGACTGGTGGTGGTATGACTGTCGGTTTGCATGATGTTGTCTTGTTGATTAAGAAAATAGGTGACCTAGACTTCAGCGACCGTGAAAAGGTTTTGGATGAATTACTAGACTACCATTTCGAAAGAAAGAGTTACGATTCCGTTATTAACGTTTTGTCAGTGGCTTTGTATTCTTTGTTCGCTGCTGACAGCGATAACTTGAAGGCATTACAAAAAGGTTGTTTCAAATATTTCCAAAGAGGTGGCGATTGTGTCAACAAACCCGTTGAATTTCTGTCTGGTGTCTTGCCAAAGCCTTTGCAATTGACCAGGGTTTTCTTCGCTGTCGCTTTTTACACCATTTACTTGAACATGGAAGAACGTGGTTTCTTGGGATTACCAATGGCTTTATTGGAAGGTATTATGATTTTGATCACAGCTATTAGAGTATTCACCCCATTTTTGTTTGGTGAGTTGATTGGTTAA |
| *UPC2.1* | AGGAGCAACAGTAGTGAAGTATGTCACGAGAAAGGCAGACGGTAGCGTGGAGTCTGATTCATCGGTAGATTTACCTCCTACGATCAAGAAGGAGCAGACACCGTTCAATGATATCCAATCAGCGGTAAAAGCTTCAGGCTCATCCAATGATTCCTTTCCATCAAGCGCCTCTACAACTAAGAGTGAGAGCGAGGAAAAGTCATCGGCCCCTATAGAGGACAAAAACAATATGACTCCTCTAAGTATGGGCCTCCAGGGTACCATCAATAAGAAAGATATGATGAATAACTTTTTCTCTCAAAATGGCACTATTGGTTTTGGTTCTCCTGAAAGATTGAATTCAGGTATCGATGGCTTACTATTACCGCCATTGCCTTCTGGAAATATGGGTGCGTTCCAACTTCAGCAACAGCAGCAAGTGCAGCAGCAATCTCAACCACAGACCCAAGCGCAGCAAGCAAGTGGAACTCCAAACGAGAGATATGGTTCATTCGATCTTGCGGGTAGTCCTGCATTGCAATCCACGGGAATGAGCTTATCAAATAGTCTAAGCGGGATGTTACTATGTAACAGGATTCCTTCCGGCCAAAACTACACTCAACAACAATTACAATATCAATTACACCAGCAGCTGCAATTGCAACAGCATCAGCAAGTTCAGCTGCAGCAGTATCAACAATTACGTCAGGAACAACACCAACAAGTTCAGCAACAACAACAGGAACAACTCCAGCAATACCAACAACATTTTTTGCAACAGCAGCAACAAGTACTGCTTCAGCAAGAGCAACAACCTAACGATGAGGAAGGTGGCGTTCAGGAAGAAAACAGCAAAAAGGTAAAGGAAGGGCCTTTACAATCACAAACAAGCGAAACTACTTTAAACAGCGATGCTGCTACATTACAAGCTGATGCATTATCTCAGTTAAGTAAGATGGGGCTAAGCCTAAAGTCGTTAAGTACCTTTCCAACAGCTGGTATTGGTGGTGTTTCCTATGACTTTCAGGAACTGTTAGGTATTAAGTTTCCAATAAATAACGGCAATTCAAGAGCTACTAAGGCCAGCAACGCAGAGGAAGCTTTGGCCAATATGCAAGAGCATCATGAACGTGCAGCTGCTTCTGTAAAGGAGAATGATGGTCAGCTCTCTGATACGAAGAGTCCAGCGCCATCGAATAACGCCCAAGGGGGAAGTGCTAGTATTATGGAACCTCAGGCGGCTGATGCGGTTTCGACAATGGCGCCTATATCAATGATTGAAAGAAACATGAACAGAAACAGCAACATTTCTCCATCAACGCCCTCTGCAGTGTTGAATGATAGGCAAGAGATGCAAGATTCTATAAGTTCTCTAGGAAATCTGACAAAAGCAGCCTTGGAGAACAACGAACCAACGATAAGTTTACAAACATCACAGACAGAGAATGAAGACGATGCATCGCGGCAAGACATGACCTCAAAAATTAATAACGAAGCTGACCGAAGTTCTGTTTCTGCTGGTACCAGTAACATCGCTAAGCTTTTAGATCTTTCTACCAAAGGCAATCTGAACCTGATAGACATGAAACTGTTTCATCATTATTGCACAAAGGTCTGGCCTACGATTACAGCGGCCAAAGTTTCTGGGCCTGAAATATGGAGGGACTACATACCGGAGTTAGCATTTGACTATCCATTTTTAATGCACGCTTTGTTGGCATTCAGTGCCACCCATCTTTCGAGGACTGAAACTGGACTGGAGCAATACGTTTCATCTCACCGCCTAGACGCTCTGAGATTATTAAGAGAAGCTGTTTTAGAAATATCTGAGAATAACACCGATGCGCTAGTTGCCAGCGCCCTGATACTAATCATGGACTCGTTAGCAAATGCTAGTGGTAACGGCACTGTAGGAAACCAAAGTTTGAATAGCATGTCACCAAGCGCTTGGATCTTTCATGTCAAAGGTGCTGCAACAATTTTAACCGCTGTGTGGCCTTTGAGTGAAAGATCTAAATTTCATAACATTATATCTGTTGATCTTAGCGATTTAGGCGATGTCATTAACCCTGATGTTGGAACAATTACTGAATTGGTATGTTTTGATGAAAGTATTGCCGATTTGTATCCTGTCGGCTTAGATTCGCCATATTTGATAACACTAGCTTATTTAGATAAATTGCACCGTGAAAAAAACCAGGGTGATTTTATTCTGCGGGTATTTACATTTCCAGCATTGCTAGACAAGACATTCCTGGCATTACTGATGACAGGTGATTTAGGTGCAATGAGAATTATGAGATCATATTATAAACTACTTCGAGGATTTGCCACAGAGGTCAAGGATAAAGTCTGGTTTCTCGAAGGAGTCACGCAGGTGCTGCCTCAAGATGTTGACGAATACAGTGGAGGTGGTGATATGCATATGATGCTAGATTTCCTCGGTGGCGGATTACCATCGATGACAACAACAAATTTCTCTGATTTTTCGTTATGA |
| *POS5* | ATGTTTGTCAGGGTTAAATTGAATAAACCAGTAAAATGGTATAGGTTCTATAGTACGTTGGATTCACATTCCCTAAAGTTACAGAGCGGCTCGAAGTTTGTAAAAATAAAGCCAGTAAATAACTTGAGGAGTAGTTCATCAGCAGATTTCGTGTCCCCACCAAATTCCAAATTACAATCTTTAATCTGGCAGAACCCTTTACAAAATGTTTATATAACTAAAAAACCATGGACTCCATCCACAAGAGAAGCGATGGTTGAATTCATAACTCATTTACATGAGTCATACCCCGAGGTGAACGTCATTGTTCAACCCGATGTGGCAGAAGAAATTTCCCAGGATTTCAAATCTCCTTTGGAGAATGATCCCAACCGACCTCATATACTTTATACTGGTCCTGAACAAGATATCGTAAACAGAACAGACTTATTGGTGACATTGGGAGGTGATGGGACTATTTTACACGGCGTATCAATGTTCGGAAATACGCAAGTTCCTCCGGTTTTAGCATTTGCTCTGGGCACTCTGGGCTTTCTATCACCGTTTGATTTTAAGGAGCATAAAAAGGTCTTTCAGGAAGTAATCAGCTCTAGAGCCAAATGTTTGCATAGAACACGGCTAGAATGTCATTTGAAAAAAAAGGATAGCAACTCATCTATTGTGACCCATGCTATGAATGACATATTCTTACATAGGGGTAATTCCCCTCATCTCACTAACCTGGACATTTTCATTGATGGGGAATTTTTGACAAGAACGACAGCAGATGGTGTTGCATTGGCCACTCCAACGGGTTCCACAGCATATTCATTATCAGCAGGTGGATCTATTGTTTCCCCATTAGTCCCTGCTATTTTAATGACACCAATTTGTCCTCGCTCTTTGTCATTCCGACCACTGATTTTGCCTCATTCATCCCACATTAGGATAAAGATAGGTTCCAAATTGAACCAAAAACCAGTCAACAGTGTGGTAAAACTTTCTGTTGATGGTATTCCTCAACAGGATTTAGATGTTGGTGATGAAATTTATGTTATAAATGAGGTCGGCACTATATACATAGATGGTACTCAGCTTCCGACGACAAGAAAAACTGAAAATGACTTTAATAATTCAAAAAAGCCTAAAAGGTCAGGGATTTATTGTGTCGCCAAGACCGAGAATGACTGGATTAGAGGAATCAATGAACTTTTAGGATTCAATTCTAGCTTTAGGCTGACCAAGAGACAGACTGATAATGATTAA |
| *Pd*FRS  *Mi*FRS  *Tw*OSC1^T502E^  (Codon-optimized for *S. cerevisiae*) | ATGTGGAGGCTAAAGATTGCAGAGAAAGGGAACAACCCTTACATTTTCACCACGAATGAGTATGCTGGAAGACAGATATGGGAATATGATCCTAATGCCGGAACTCCTGAAGAGCGAGAGCAAGTGGAAGAGGCTCGCCGGAACTTTACTAAGAACCGCTCTAAGGTCAAGCCCAGCTCCGACCTTCTTTGGCAATATCAGATTCTGAGGGAGAAAAACTTCAAGCAAACAATTCCGGCAGTGAGAGTTGAGGAAGGTGAAGAAGTCACGTACGAAAAGACAACAACGGCGATGAAGAGGTCAGCCAGCTTTTATTCAGCCTTGCAGGCTAGTGACGGCCACTGGCCTGCTGAGAATTCCGGGGTCCTCTTTTTCCTTCCTCCTTTCGTGTTTTGCTTCTACATTACTGGGCATCTCAACACCATGTTCCCTCCTGAGTACCGCAAAGAAATCTTTCGTTACATATACAACCATCAGAATGAAGATGGTGGGTGGGGTCTGCACATAGAAAGTCACAGCAACATGTTTTGCACAACCTTCAGCTACATTTGTCTACGTATGCTTGGGGTAGGGCCAGATGAGGAAGCTTGTGCAAGAGGAAGAAAATGGATTCTTGACCGTGGTGGTGTCACTTCCATTCCCTCCTGGGGCAAGACTTGGCTTTCGATTCTTGGCCTGTTTGACTGGTCTGGTTGTAACCCTATGCCCCCAGAGTTTTGGATTCTGCCTACTGCCCTTCCTGTTCATCCAGCAAAAATGTGGTGCTACTGTCGATTGGTTTACATGCCAATGTCATATCTGTATGGAAAAAGATTTGTTGGACCAATTACACCTCTGATTCTGTCATTGAGAGAAGAGCTGTACCTTCAGCCTTACGAAAGCGTAAAGTGGAAGCAAGTCAGACATTTATGTGCAGAGGAGGATCTTTACTATCCACATTCCTTGATTCAGGATTTCCTTTGGGATAGTCTGTATTTGATGTCTGAACCTCTTCTTACTCGCTGGCCCTTCAACCAGTTGGTCAGAAAGAAGGCTCTTGAAGTAACAATGAAGCACATTCATTATGAAGATGAAAACAGTAGATACATTACTATTGGTTGTGTTGAGAAGGTGCTGTGCATGCTTTCTTGTTGGGTGGAAGATCCAGATGGTGTTGCTTTTAAGAGGCATCTTGCTAGGGTTCCTGATTACTTGTGGGTTGGAGAGGATGGAATGAAGGTCCAGAGCTTTGGTAGCCAGTTGTGGGATGCTACTTTTGGTTTTCAGGCTTTGTATACTAGTGAGCTTGGTGAAGAAATCAAGCCGACGCTCGCTAAAGCATTTGATTTCATCAAGAAATCTCAGGTTGTGGATAATCCAGCAGGTGACTTCGTGGGCATGTATCGTCACATTTCTAAAGGATCATGGACTTTCTCTGATCAGGATCATGGCTGGCAACTTTCTGATTGTACAGCCGAAGCTTTAAAGTGTGTTCTCTTCGCTCAAATGTTGCCCACTGAATACATTGGTGAGAAAATGGATCCTCAGATGATATTTGAAGCAGTCAATATCATTCTTTCACTGCAGGGACCAAGAGGTGGTCTAGCAGGCTGGGAGCCGATTCGCGGTGAAATGTGGTTAGAGAAACTCAATCCTATGGAATTCCTGGAGAACATAGTCATCGAGCACGACTATGTTGAGTGCACATCATCTGCAATCCATGGATTTGTGATGTTTATGAAGATGTACCCTGGACACAGGAAGAAAGAAATTGAGACTTTCATTGCAAGAGCTGTCGATTATCTCGAAATGATTCAAATGCCTGATGGTTCATGGTATGGAAACTGGGGAGTTTGCTTCATTTACAGTACATGGTTCGCACTTGTTGGTCTAGCAGCTGCTGGAAAAACCTACTATAATAACCAAGCAATGCGTAGAGGAGTCGATTTTCTACTCAGGGCACAGTCTCCAGATGGTGGATGGGGCGAGAGCTACTTATCTTGCCCAAATAAGATATATACACCTCTCGAAGAAAAGAGATCAACTTATGTGCAGACAGGATGGGCTATGCTTGGTTTGATTCATTCTGGCCAGGCGGATAGGGATCCGACTCCACTTCACCGTGGTGCTAAGTTGTTGATTAACTCTCAAGCTGAAGATGGAAGCTATCCTCAGCAGGAAATTACTGGTGTTTTCAAGAATAACTGTATGTTACACTATCCAACATATAAGAATGTTTTCCCCTTATGGGCTCTGGCAGAATACCGCAAAAATGTTCCATTGCCTTCCAAGAAGCTTTGA  ATGTGGAAGATAAAGATTGCGGACCGTGGGAACTGTCCCTACAACGAGTACTTGTACACAACAAA  TGACTTTGTTGGCAGGCAGATATGGGAGTTTGACCCCAATTCAGGCACCCCTGAAGAGCTCGCTGAGATCGAAGAGGCTCGCCGAAAATTCACTGAGAATCGCTATGAAGTCAAGCCTGCTTCCGACCTCCTTTGGATGATGCAGTTTCTGAGAAAGAATAATTTCAAGCAAACAATTCCTCCCTTAAGAATTGGTGAGAAAGAACAAGTTACATATGAAGATGTCACAACTGCTTTGAGGAGAGCTTCCTCTTTCTTTTCTGCCTTGCAGGCCAGTGATGGTCACTGGCCAGCAGAAAATGCTGGTGTCTCGTTCTTCCTTCCTCCATTTATCTTTTGCCTATACATCACTGGACATCTCAATTCGATTATCACTCCGGAACATCGCAAAGAAATCCTACGTTTCATATACAACCACCAGAATGAAGATGGAGGTTGGGGAATACACATAGAGGGACACAGCACAGTGTTTGCCACAGCATTCACTTATGTTTGCATGAGAATACTTGGAGTGGGACCAGACGAAGATGCTTGTGCTAGAGCAAGAAAATGGATTCTTGATCGTGGCGGTATCACTTACATGGCTTCCTGGGGAAAGACTTGGTTTTCGGTACTTGGAATATTTGATTGGTATGGTTGCAACCCAATGCCCCCAGAGTTCTGGATTCTTCCTTCTTATCTCCCCATACATCCAGCAAAAATGTGGTGCTACTGCCGGATGGTTTACATGCCAATGTCATATCTATACGGGAAAAGATTTGTGGCTCCAATCACCCCTCTTATTTTACAATTGAGAGAAGAGCTCCACACTCAGCCCTACCATGAAATTGAATGGAGAAAAATGCGGCATCGATGCGCAGAGGAGGATCTGTACTTTCCCCACAGTCTGATCCAGAATTTCCTCTGGGACAGTCTTTATGTGGCTTCTGAACCTCTTTTGACTCGATGGCCTTTTAGCAAGATAAGAGAGAGGGCTCTTGAAAAAGCAATGGAACACATTCACTATGAAGATGAGAATAGTCGATACATCACCATCGGTTGCGTTGAAAAGGCGCTGTGTATGCTTTGTTGTTGGGTTGAAGATCCCAATGGAGAATATTTCAAGAAGCATCTTGCAAGGATACCAGATTATTTATGGGTTGCTGAGGATGGTATGAAAGTGGTGAGTTTTGGCAGCCAACTGTGGGATGCAACCTTTGGCTTTCAAGCCCTGGTTGCTAGCAATCTCACAGATGAAGTAGCTCCTACTCTTGTCAAAGCATACGACTTCATAAAGAAATGTCAGGTCAGAGATAACCCCTCTGGCAACTTTGAGAAAATGTTTCGCCACATTTCCAAAGGATCGTGGACTTTCTCTGATCAAGATCATGGATGGCAACTTTCTGATTGCACTGCTGAAGCCTTAAAGTGCTGCCTGCTAGCAGCAACCATGCCTGAAGAGCTTGTCGGTGAGAAATTAGATCCTCAATGGATATTTGAGTCTGTAAACATCATACTTTCCCTCCAAGAGCCTAAAACTGGTGGTTTAGCCGGCTGGGAACCAGTAAGAGCTGGACAATGGATGGAGATGCTCAACCCTATGGAGTTTCTTGAGAATATTGTGATTGAACATACATATATTGAGTGCACTGGATCTTCAATCATTGCTTTCATTACACTCAAGAAGTTATTCCCAGGTCATAGGACCAAAGACATTGACAATTTCATTGTAAACGCCATAAGATATCTTGAGGATGAGCAGTATCCTGATGGCTCGTGGTATGGGAACTGGGGTATTTGCTTCATCTATAGTACAATGTTTGCACTCGGAGGGCTAGCAGCAGCTGGTAGAACTTACAAAAACTGTCAGGCTGTGCGCAGAGGTGTTGACTTTCTACTAATTAATCAGAGTGACGACGGGGGATGGGGAGAAAGCTATATTTCTTGCCCAAGAAAGAAATATACGCCTCTTGAAGGAAGAAGATCAAATGTTGTACAAACTGCATGGGCTATGCTAGGGCTGCTCTATGCTGGCCAGGCTGAGAGAGATCCCACTCCTCTCCACCGTGGTGCGAAGTTACTAATCAATTACCAAATGGAAGAGGGAGGTTACCCTCAGCAGGAAATCACTGGAGTTTTCAAAATGAATTGCATGTTACACTATCCTATATATAGGAATGCTTTCCCAATATGGGCGCTCGGAGAGTACCGGAAGCGTGTTCCATTGCCTTCCAAAGGAAATTCAATGGCAATGAAAATAAATTCAGCTTAA  ATGTGGAAGTTGAAAGTTGCCGAAAGAGGTAACGCTCCATATTCTGAATACTTGTACACTACCAACGACTTCTCTGGTAGACAAACTTGGGAATTTGATCCAAATGCTGGTACTCCACAAGAATTGGCTAAAGTTGAAGAAGCCAGAAGAAAGTTCACCGAAGATAGACATACTGTTAAGCCAGCTTCAGATTTGTTGTGGATGATGCAATTCATGCGTGAGAAGAACTTCAAGCAAACTATTCCACCAGTTAGGTTGGGTGAAGAGGAACAAGTTACTTACGAAGATTTGACTACTGCTTTGACTAGGACTACCAATTTCTTTACCGCTTTACAAGCTTCTGATGGTCATTGGCCAGCTGAAAATGGTGGTGTTTCTTTTTTCTTGCCACCATTCATCTTCAGCTTGTACATTACTGGTCACCTGAACTCTATTATTACCCCAGAGTACAGAAAAGAGATCCTGAGATTCATCTACAACCACCAAAATGAAGATGGTGGTTGGGGTATTCATATCGAAGGTCATTCTACTATGTTCGGTACTGCTTTCTCTTACGTCTGCTTGAGAATTTTGGGTATCGAAGTTGATGGTGGTAAGGATAATGCTTGTGCTAGAGCTAGAAAGTGGATTTTGGATCATGGTGGTATTACCTATATGCCATCTTGGGGTAAAACCTGGTTGTCTATTTTGGGTGTTTATGATTGGTACGGCTGTAATCCAATGCCACCTGAATTTTGGTTGTTGCCATCTTACTTGCCAATTCATCCAGCTAAGATTTGGTGTTACTGCAGAATGGTTTACATGCCCATGTCTTACTTGTACGGTAAAAGATTCGTTGGTCCAATCACTCCATTGATCTTGCAATTGAGAGAAGAATTGCATACCCAACCATTCCACGAAATTCAATGGCGTCAAACTAGACATAGATGTGCTAAAGAGGACTTGTACTACCCACACTCTTTGATTCAAGATTTCATCTGGGATTCCTTGTACGTTGCTTCTGAACCATTATTGACTAGATGGCCATTGAACAAGATTAGAGAAAAGGCTTTGGCTAAGGCCATGGAACATATTCATTATGAGGACGAAAACTCCAGGTACATTACCATTGGTTGTGTTGAAAAAGCCTTGTGTATGTTGTGTTGCTGGGTTGAAGATCCAAACTCTGACTACTTCAAGAAACACTTGGCTAGAATTCCAGATTACTTGTGGGTTGCCGAAGATGGTATGAAGGTTCAATCTTTTGGTTCCCAATTGTGGGATGCTACTTTTGGTTTTCAAGCTTTGGTTGCTTCTAACTTGACCGAAGATGAAGTTGGTCCAGCTTTGGCAAAAGCTTACGATTTCATTAAGAAGTCCCAAGTCAAGGATAACCCATCTGGTGATTTTGAATCCATGCATAGGCATATCTCTAAAGGTTCTTGGACCTTCTCTGATCAAGATCATGGTTGGCAATTGTCTGATTGCACTGCTGAAGCTTTGAAGTGTTGTTTGTTGGCTGCTGAAATGCCACAAGAAGTTGTTGGAGAAAAAATGAAGCCTGAATGGGTTTACGAAGCCATCAACATTATCTTGTCCTTGCAATCTAAATCCGGTGGTTTGGCTGGTTGGGAACCTGTTAGAGCTGGTGAATGGATGGAAATTTTGAACCCAATGGAATTCTTGGAGAACATCGTTATCGAACACACCTACGTTGAATGTACCGGTTCTTCTATTATTGCCTTCGTGTCTCTGAAAAAGTTGTACCCAGGTCATAGAACTAAGGACATCGATAACTTCATTAGAAACGCCATCAGGTACTTGGAAGATGTTCAATATCCAGATGGTTCTTGGTATGGTAACTGGGGTATTTGTTTCATCTACTCTACCATGTTTGCCTTAGGTGGTTTAGCTGCTACTGGTAGAACTTACGATAATTGCCAAGCTGTTAGAAGAGGTGTCGACTTCATATTGAAGAACCAGTCTGATGACGGTGGATGGGGTGAATCTTATTTGTCTTGTCCAAGAAAGGTTTACACCCCATTGGATGGTAGAAGATCTAACGTTGTTCAAACTGCTTGGGCTATGTTGGGTTTGTTGTATGCTGGTCAAGCTGAAAGGGATCCAACTCCATTGCATAGAGGTGCAAAAGTTTTGATCAACTACCAGATGGAAGATGGCGGTTACCCACAACAAGAAATTACTGGTGTTTTCAAGATGAACTGCATGTTGCATTACCCCATCTACAGAAATGCTTTTCCAATTTGGGCTTTGGGTGAATACCGTAAAAGAGTTCCATTGCCATCTAAGGGTTACTAA |
| 15site in  ChrXVI | ATAAAGCAGCCGCTACCAAACAGACAAGATTCAGTATGTAAGGTAAATACCTTTTTGCACAGTTAAACTACCCAAACTTATTAAAGCTTGATAAATTACTGAAATTCCACCTTTCAGTTAGATTCAGGCCTCATATAGATTAGATATAGGGTACGTAACATTCTGTCAACCAAGTTGTTGGAATGAAAGTCTAAAATGTCATCTATTCGGTAGCACTCATGTTACTAGTATACTGTCACATGCGGTGTAACGTGGGGACATAAAACAGACATCAAATATAATGGAAGCTGAAATGCAAAGATCGATAATGTAATAGGAATGAAACATATAAAACGAAAGGAGAAGTAATGGTAATATTAGTATGTAGAAATACCGATTCAATTTTGGGGATTCTTATATTCTCGAGAGAATTTCTAGTATAATCTGTATACATAATATTATAGGCTTTACCAACAATGGAATTTCGACAATTATCATATTATTCACCAATTAATCACAAGTTGGTAATGAGTTTGATAACAAGTTACTTTCTTAACAACGTTAGTATCGTCAAAACACTCGGTTTTACTCGAGCTTGTAGCACAATAATACCGTGTAGAGTTCTGTATTGTTCTTCTTAGTGCTTGTATATGCTCATCCCGACCTTCCATTTTTTTTTTCTTGGAATCAGTACATAGCAGGTATGAGTTGTTAGAGCTGTTACAAGTTACGGTAAACATTTCAACACACCGTTATTTAACGAATTTATTTGAGAAAGTGGTGTATTTTAAGATATATGTTTGGTTTCGATTGTTGGCAAAGACTATAATATTATGCATATAGGATATACCAAAAATTCTCTCTGAGGATATAGGAATCTACAAAATGAATCTACATTTC |
| 15site1    15site2  15site1-  His3-L1  L1  L2  L3  L4 | ATAAAGCAGCCGCTACCAAACAGACAAGATTCAGTATGTAAGGTAAATACCTTTTTGCACAGTTAAACTACCCAAACTTATTAAAGCTTGATAAATTACTGAAATTCCACCTTTCAGTTAGATTCAGGCCTCATATAGATTAGATATAGGGTACGTAACATTCTGTCAACCAAGTTGTTGGAATGAAAGTCTAAAATGTCATCTATTCGGTAGCACTCATGTTACTAGTATACTGTCACATGCGGTGTAACGTGGGGACATAAAACAGACATCAAATATAATGGAAGCTGAAATGCAAAGATCGATAATGTAATAGGAATGAAACATATAAAACGAAAGGAGAAGTAATGGTAATATTAGTATGTAGAAATACCGATTCAATTTTGGGGATTCTTATATTCTCGAGAGAATTTCTAGTATAATCTGTATACATAATATTATAGGCTTTACCAA  CAATGGAATTTCGACAATTATCATATTATTCACCAATTAATCACAAGTTGGTAATGAGTTTGATAACAAGTTACTTTCTTAACAACGTTAGTATCGTCAAAACACTCGGTTTTACTCGAGCTTGTAGCACAATAATACCGTGTAGAGTTCTGTATTGTTCTTCTTAGTGCTTGTATATGCTCATCCCGACCTTCCATTTTTTTTTTCTTGGAATCAGTACATAGCAGGTATGAGTTGTTAGAGCTGTTACAAGTTACGGTAAACATTTCAACACACCGTTATTTAACGAATTTATTTGAGAAAGTGGTGTATTTTAAGATATATGTTTGGTTTCGATTGTTGGCAAAGACTATAATATTATGCATATAGGATATACCAAAAATTCTCTCTGAGGATATAGGAATCTACAAAATGAATCTACATTTC  ATAAAGCAGCCGCTACCAAACAGACAAGATTCAGTATGTAAGGTAAATACCTTTTTGCACAGTTAAACTACCCAAACTTATTAAAGCTTGATAAATTACTGAAATTCCACCTTTCAGTTAGATTCAGGCCTCATATAGATTAGATATAGGGTACGTAACATTCTGTCAACCAAGTTGTTGGAATGAAAGTCTAAAATGTCATCTATTCGGTAGCACTCATGTTACTAGTATACTGTCACATGCGGTGTAACGTGGGGACATAAAACAGACATCAAATATAATGGAAGCTGAAATGCAAAGATCGATAATGTAATAGGAATGAAACATATAAAACGAAAGGAGAAGTAATGGTAATATTAGTATGTAGAAATACCGATTCAATTTTGGGGATTCTTATATTCTCGAGAGAATTTCTAGTATAATCTGTATACATAATATTATAGGCTTTACCAACTAGTACACTCTATATTTTTTTATGCCTCGGTAATGATTTTCATTTTTTTTTTTCCCCTAGCGGATGACTCTTTTTTTTTCTTAGCGATTGGCATTATCACATAATGAATTATACATTATATAAAGTAATGTGATTTCTTCGAAGAATATACTAAAAAATGAGCAGGCAAGATAAACGAAGGCAAAGATGACAGAGCAGAAAGCCCTAGTAAAGCGTATTACAAATGAAACCAAGATTCAGATTGCGATCTCTTTAAAGGGTGGTCCCCTAGCGATAGAGCACTCGATCTTCCCAGAAAAAGAGGCAGAAGCAGTAGCAGAACAGGCCACACAATCGCAAGTGATTAACGTCCACACAGGTATAGGGTTTCTGGACCATATGATACATGCTCTGGCCAAGCATTCCGGCTGGTCGCTAATCGTTGAGTGCATTGGTGACTTACACATAGACGACCATCACACCACTGAAGACTGCGGGATTGCTCTCGGTCAAGCTTTTAAAGAGGCCCTACTGGCGCGTGGAGTAAAAAGGTTTGGATCAGGATTTGCGCCTTTGGATGAGGCACTTTCCAGAGCGGTGGTAGATCTTTCGAACAGGCCGTACGCAGTTGTCGAACTTGGTTTGCAAAGGGAGAAAGTAGGAGATCTCTCTTGCGAGATGATCCCGCATTTTCTTGAAAGCTTTGCAGAGGCTAGCAGAATTACCCTCCACGTTGATTGTCTGCGAGGCAAGAATGATCATCACCGTAGTGAGAGTGCGTTCAAGGCTCTTGCGGTTGCCATAAGAGAAGCCACCTCGCCCAATGGTACCAACGATGTTCCCTCCACCAAAGGTGTTCTTATGTAGCCCCGGTCCGTTTGTTCTATACTTCTCTCTGCTATACCTACAAGCAAGGTAATCGGAAGTAGTATTACGCAGGAATATCCCGCGCGAAGCTACAATTTTTGGACTCCAACGTCAAAGCAGGGGAGTCAGAAGTCCCCTCTAAAATTGCCT  CCCCGGTCCGTTTGTTCTATACTTCTCTCTGCTATACCTACAAGCAAGGTAATCGGAAGTAGTATTACGCAGGAATATCCCGCGCGAAGCTACAATTTTTGGACTCCAACGTCAAAGCAGGGGAGTCAGAAGTCCCCTCTAAAATTGCCT  GACAAAGCGCCAAGGAACTGTAATATATAGCTACGCCCTATCTGGACGATTGGGCGACTTTTACGTACGGTTGCTCAATTCCTACGCAACTTAATATATTTTGCAACGGTTAAATCGGCTTGAAGCTCGGGCTATCCAACTCGCGGACTA  AACGACGGTAGACGCCAACTACGCTGACAGACCGATTTGTTTAAGATTAGAAGATTTTTAGCCGCGCCGCAATCGGAACCAGCAAACTCAATTCTGGGAACAGTTTAAAATACTAGTAATTACGATAGCCGAGAAACGGACTAAGTCCGC  CCAGACGATACAGAGGCTAAGAATAACGCAGATAATCGCTCTAACGAAACGTACTAAAAGATTTCTTTTGAAGTAACTAGATACCCTGGTCTTATACTAGGTATCTTTGTCAGAAACGGCCTAAGACTACAGTAAGAGCAGTTGGAACCT |

**Supplementary Figures**

#
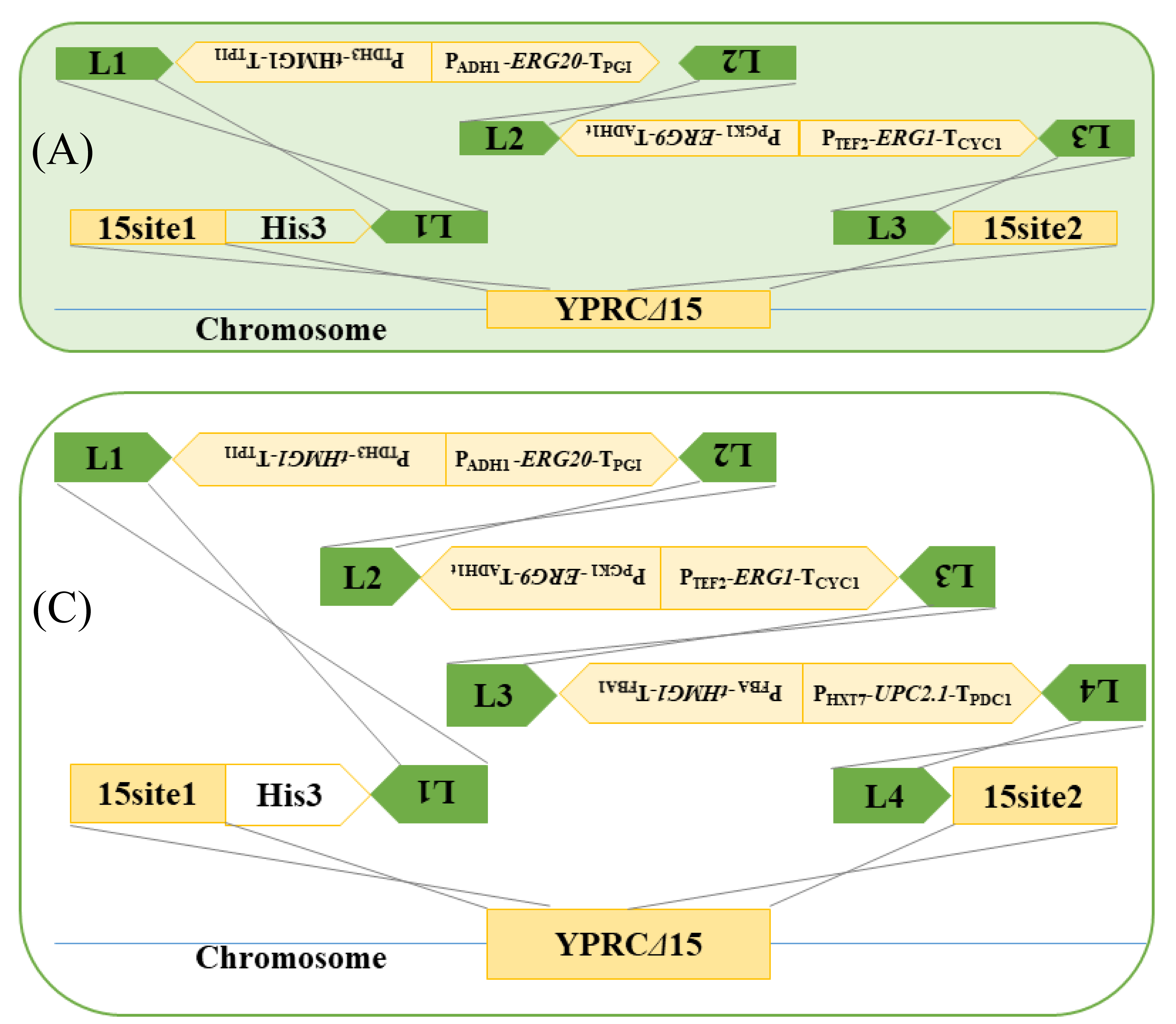


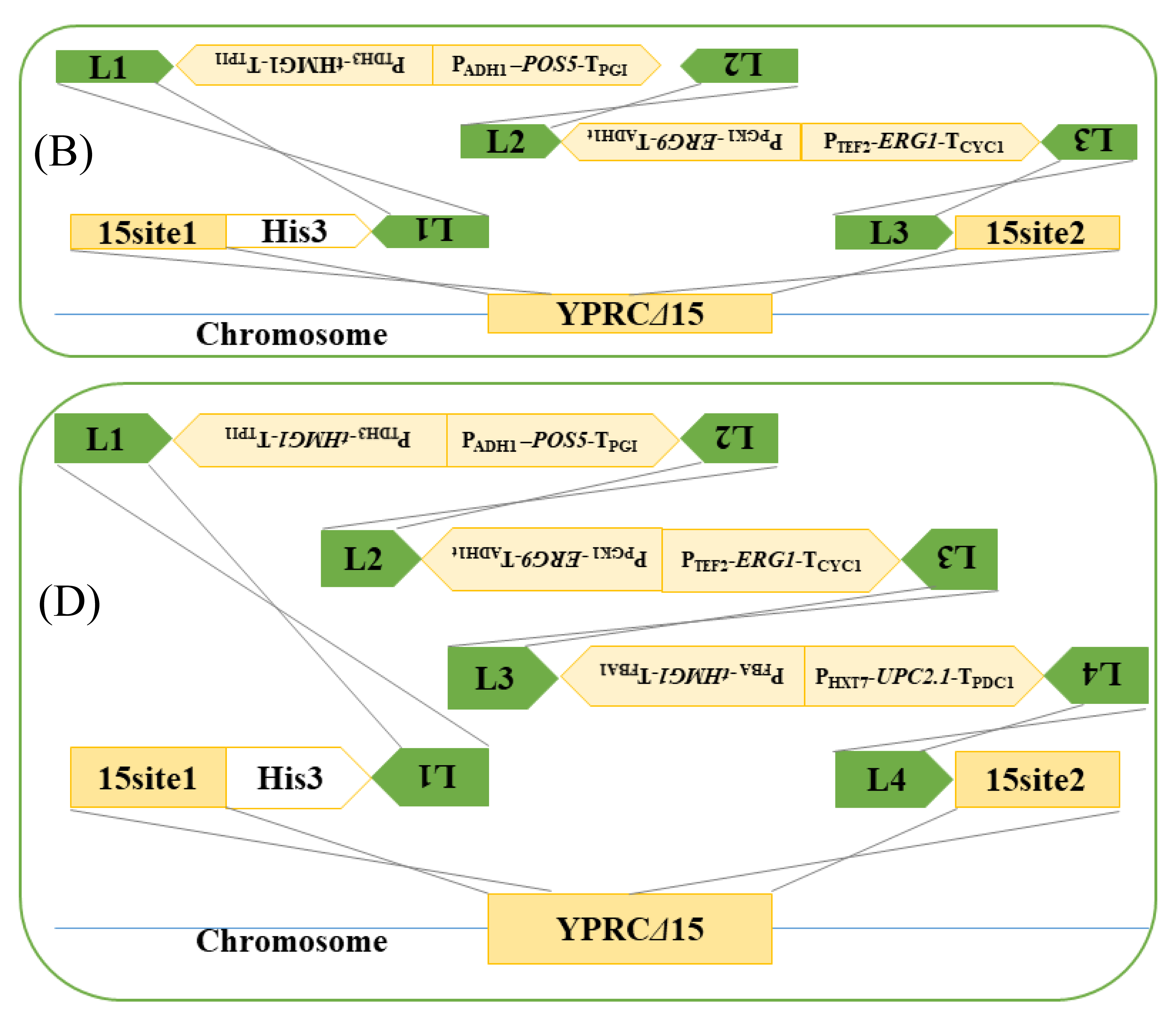


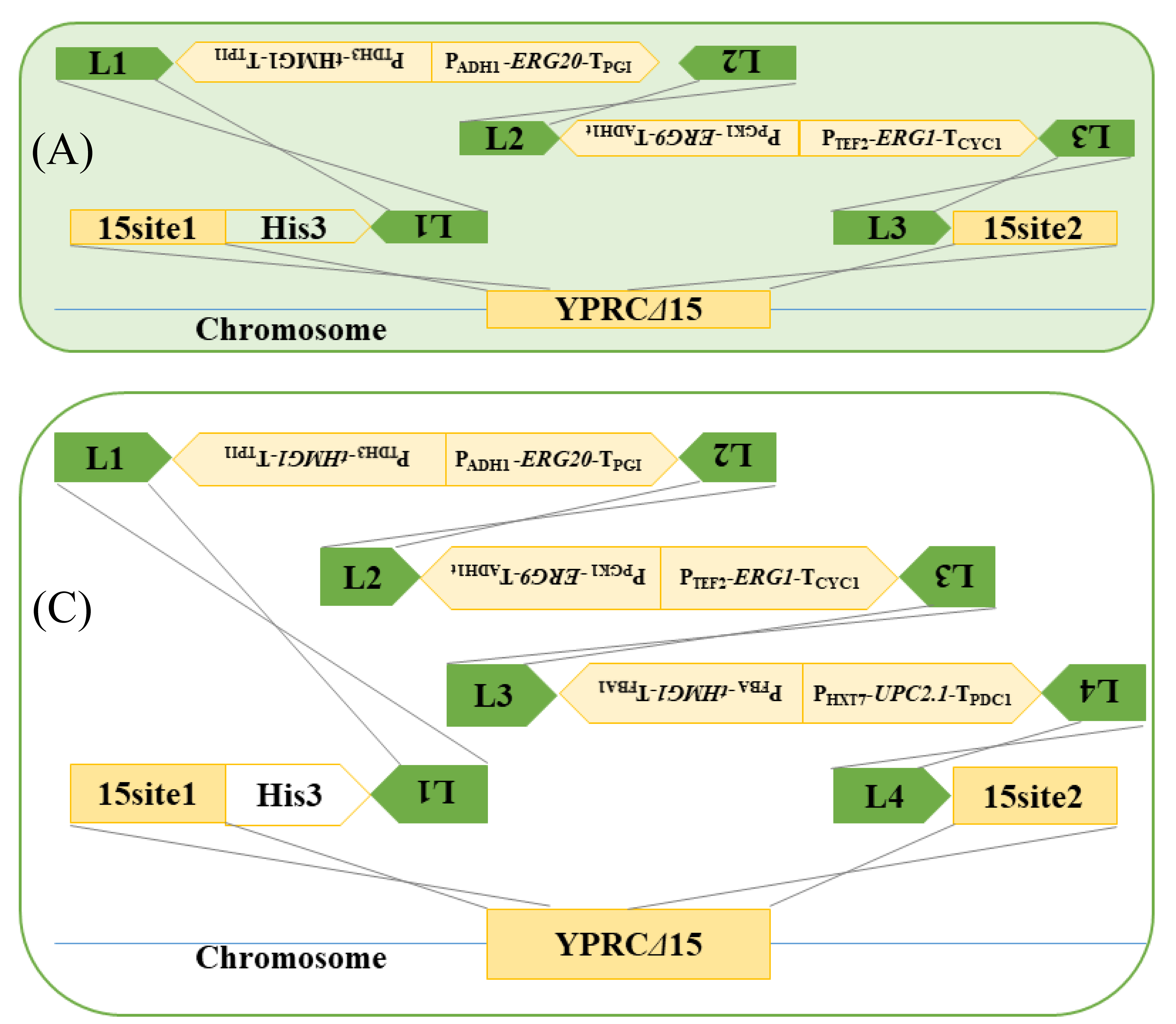


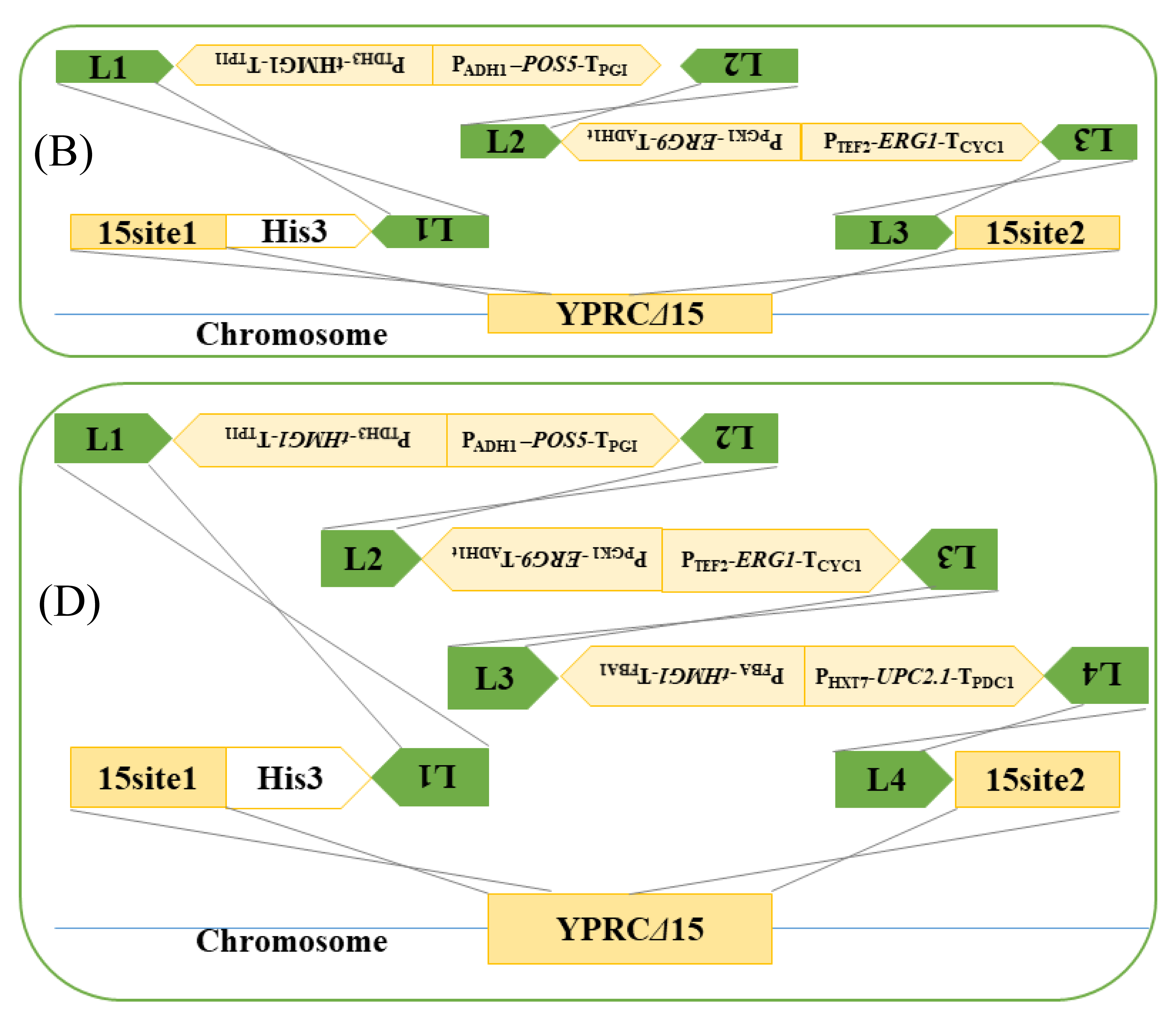


# Supplementary Figure S1. Assembly of overexpression modules and construction of head and tail homologous arms.

# The expression cassette harboring *tHMG1*/*ERG20*, *ERG9/ERG1*, and *tHMG1*/*UPC2.1* genes was integrated into *S. cerevisiae* strain BY4741 chromosome XVI-15site in different combinations and resulted in the strains GH1 (A), GH2 (B), GH3 (C), GH4 (D).

#


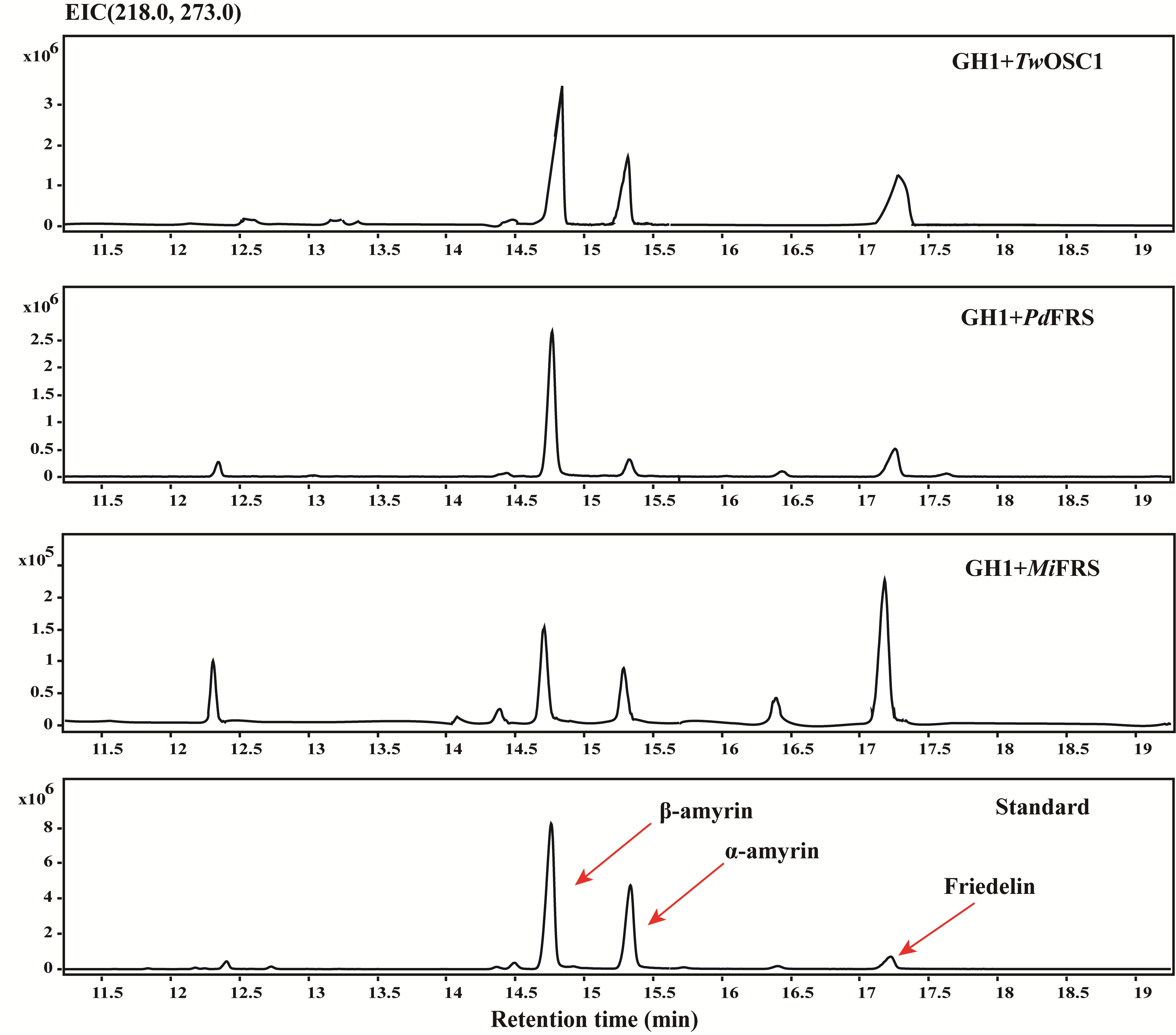


# Supplementary Figure S2. GC-MS analysis of the products in recombinant strain GH1. GH1+*Tw*OSC1: Yeast strain GH3 harbouring the *Tw*OSC1^T502E^ from *Tripterygium wilfordii*. GH1+*Mi*FRS: Yeast strain GH3 harbouring the *Pd*FRS from *Populus davidiana*. GH1+*Pd*FRS: Yeast strain GH3 harbouring the *Mi*FRS from *Maytenus ilicifolia*. Standards: *β*-amyrin, *a*-amyrin, and friedelin.

#
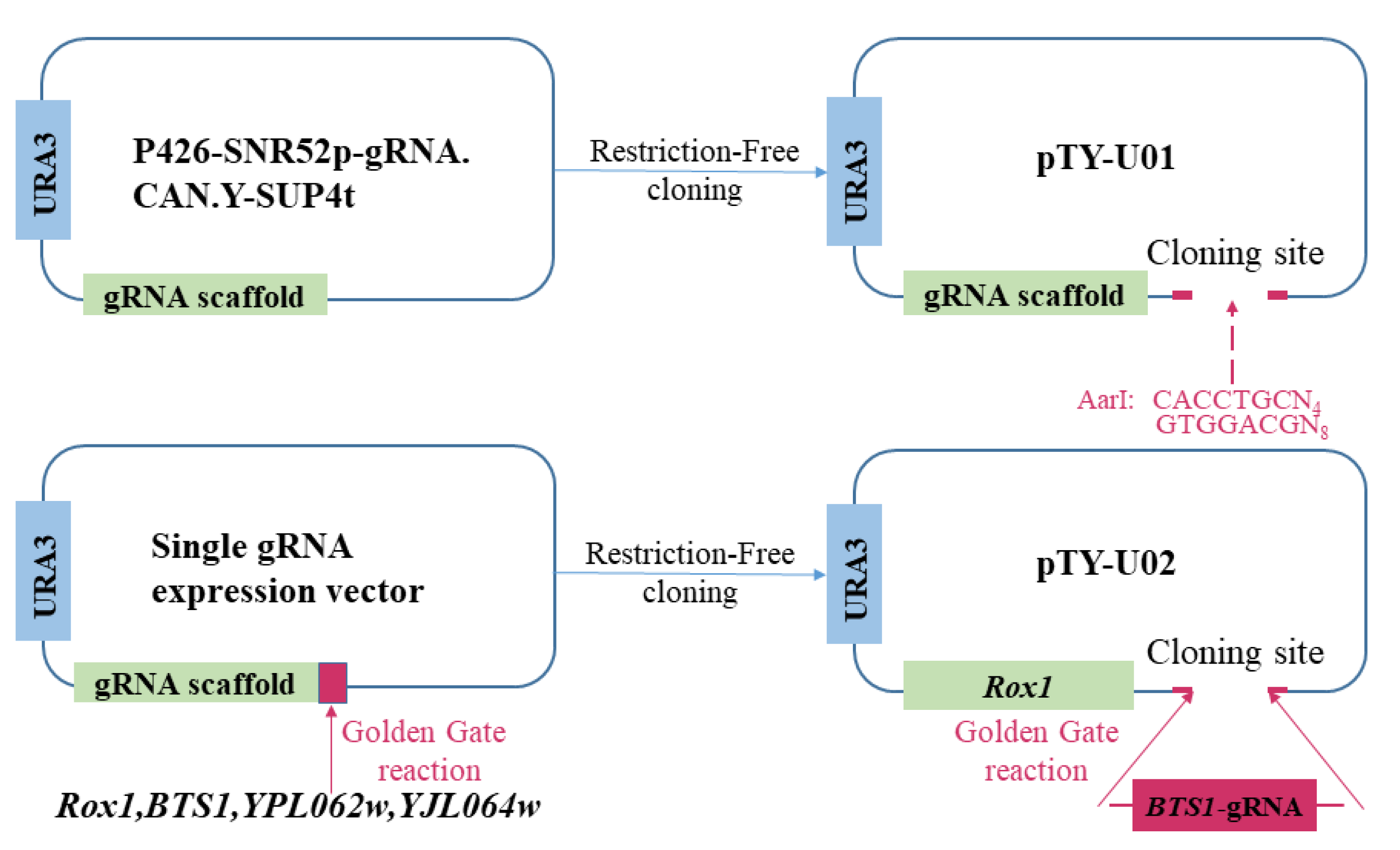


# Supplementary Figure S3. Construction of gRNA expression cassettes.

The reaction system of sgRNA oligos was performed as follows:

| **Component** | **Amount** | **Final** |
| --- | --- | --- |
| Annealing buffer, 10× | 2 μL | 1× |
| Forward oligo (10 µM) | 9 μL | 4.5 µM |
| Reverse oligo (10 µM) | 9 μL | 4.5 µM |
| Total | 20 μL |  |

Anneal the oligos in a thermocycler using the following conditions:

| **Step** | **Condition** |
| --- | --- |
| 1 | 95 °C, 5 min |
| 2 | 95–25 °C, -1 °C min-1, 70 min |
| 3 | 4 °C, Hold |

Golden Gate digestion reaction system was as follows:

| **Component** | **Amount** | **Final** |
| --- | --- | --- |
| AarI | 2 μL | 1× |
| 10×Buffer AarI | 2 μL | 4.5 µM |
| 50×oligonucleotide (0.025 mM) | 0.4 μL | 4.5 µM |
| T4 Ligase (HC) | 1 μL |  |
| T4 Ligase Buffer | 2 μL |  |
| vectors and insert fragments | depends | each 30 fmol |
| Total | 20 μL |  |


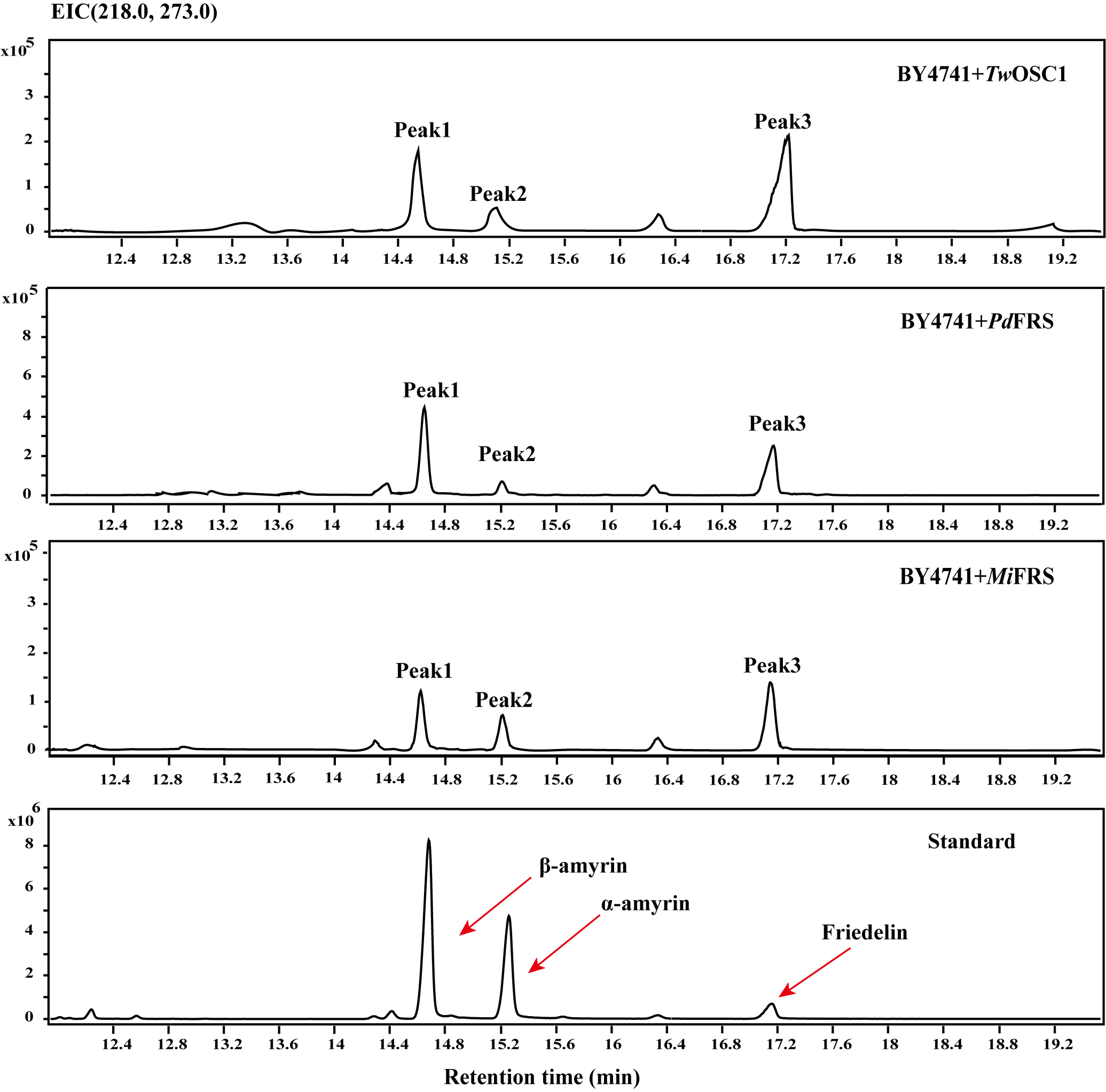


# Supplementary Figure S4. Gas chromatography-mass spectrometry (GC-MS) analysis of the products in yeast strain BY4741. BY4741+*Tw*OSC1: Yeast strain BY4741 harbouring the *Tw*OSC1^T502E^ from *Tripterygium wilfordii*. BY4741+*Mi*FRS: Yeast strain BY4741 harbouring the *Pd*FRS from *Populus davidiana*. BY4741+*Pd*FRS: Yeast strain BY4741 harbouring the *Mi*FRS from *Maytenus ilicifolia*. Standards: *β*-amyrin, *a*-amyrin, and friedelin.


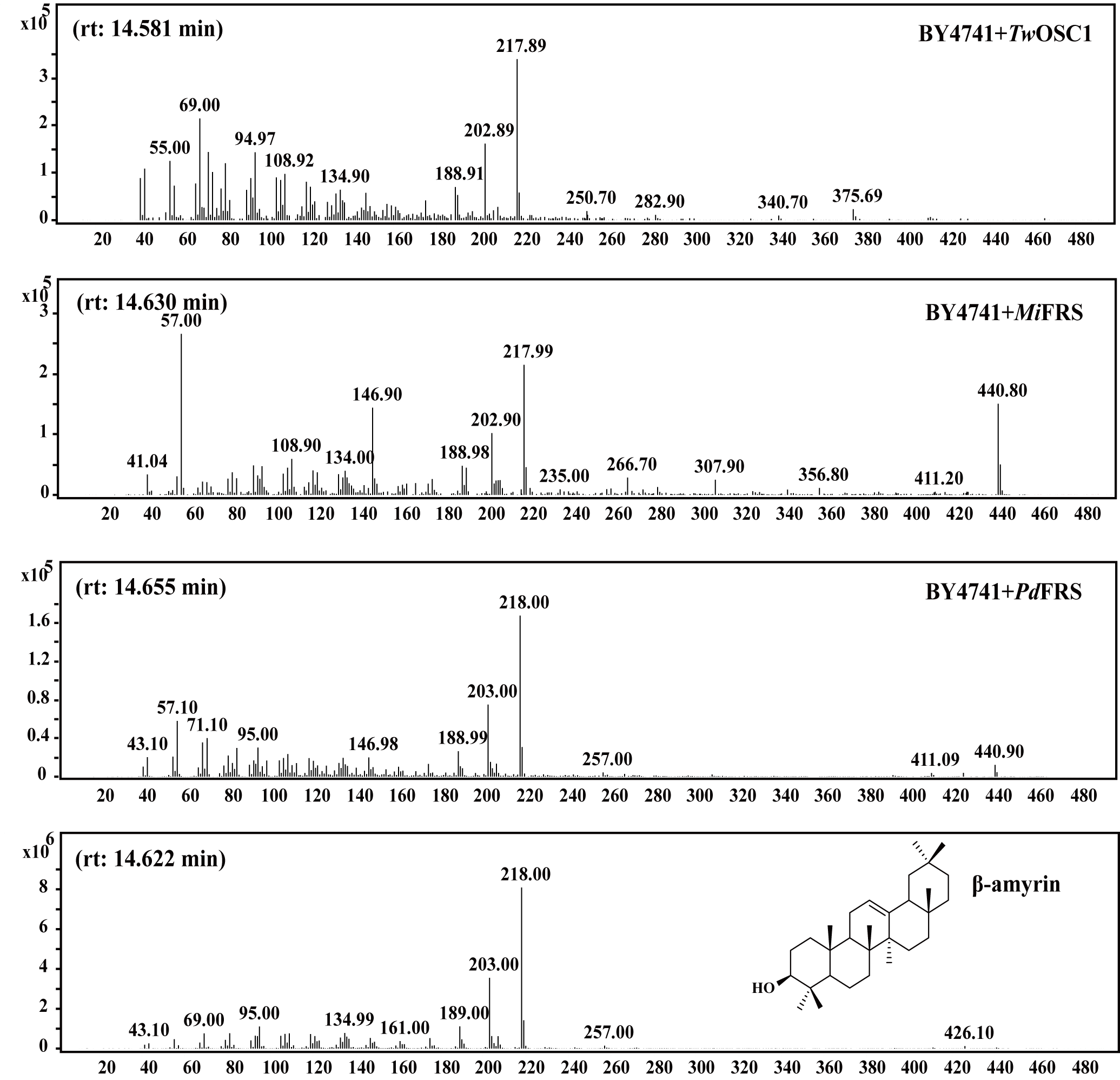


# Supplementary Figure S5. MS spectrum of extracts of *Tw*OSC1 ^T502E^, *Mi*FRS, *Pd*FRS expressed in yeast BY4741 (peak 1). Standard: *β*-amyrin.


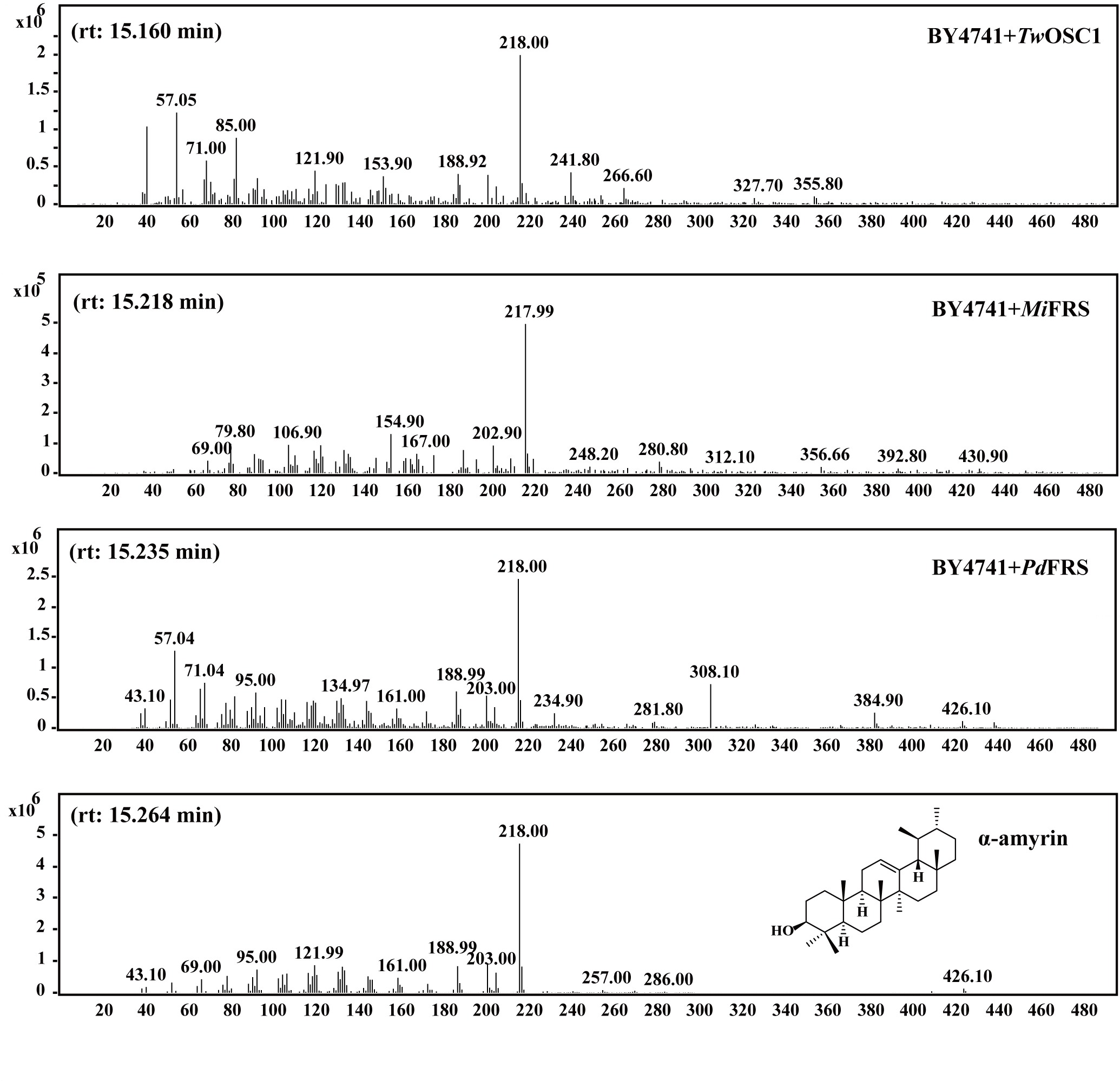


# Supplementary Figure S6. MS spectrum of extracts of *Tw*OSC1 ^T502E^, *Mi*FRS, *Pd*FRS expressed in Yeast BY4741 (peak 2). Standard: *α*-amyrin.


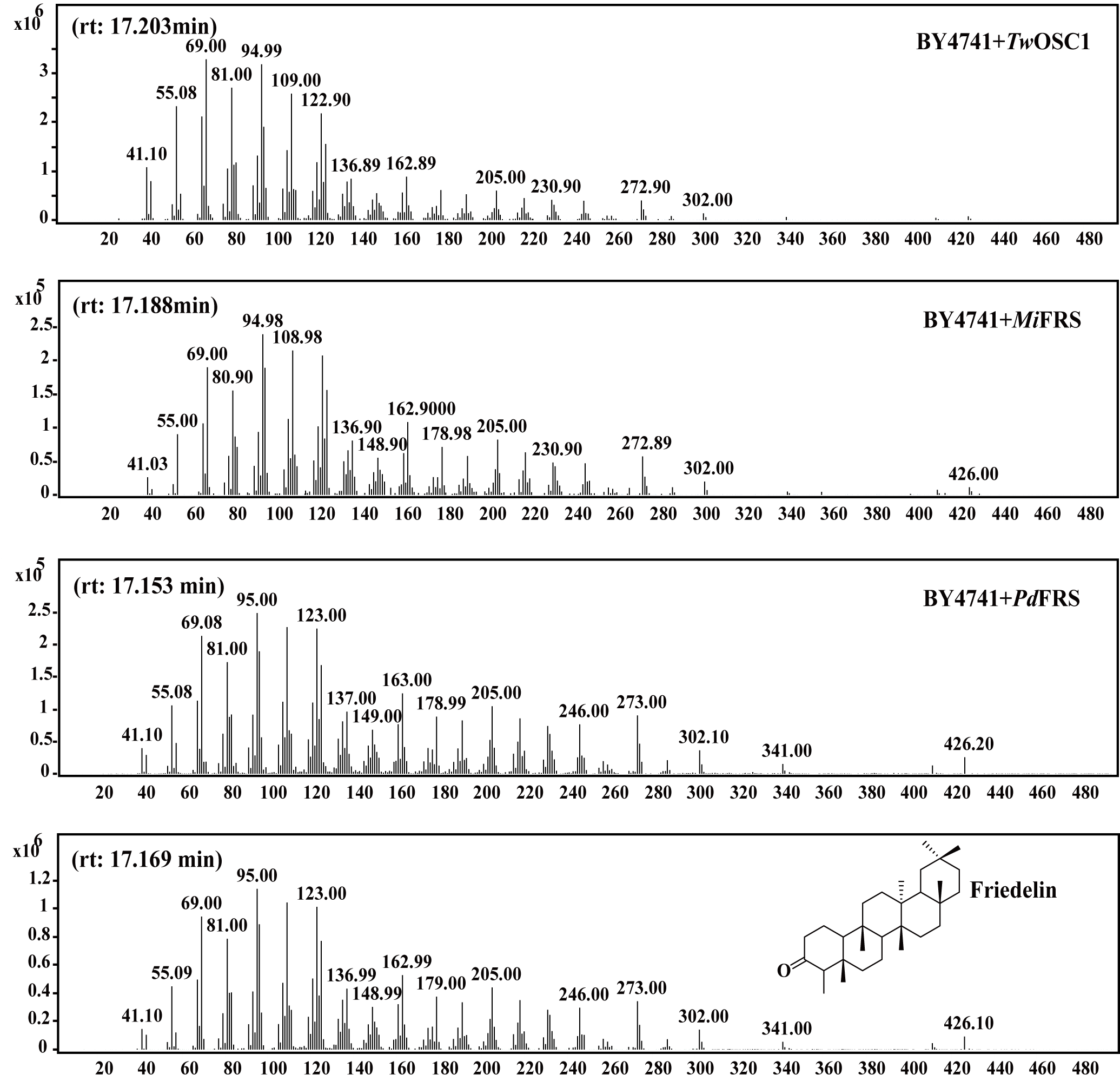


# Supplementary Figure S7. MS spectrum of extracts of *Tw*OSC1 ^T502E^, *Mi*FRS, *Pd*FRS expressed in Yeast BY4741 (peak 3). Standard: friedelin.


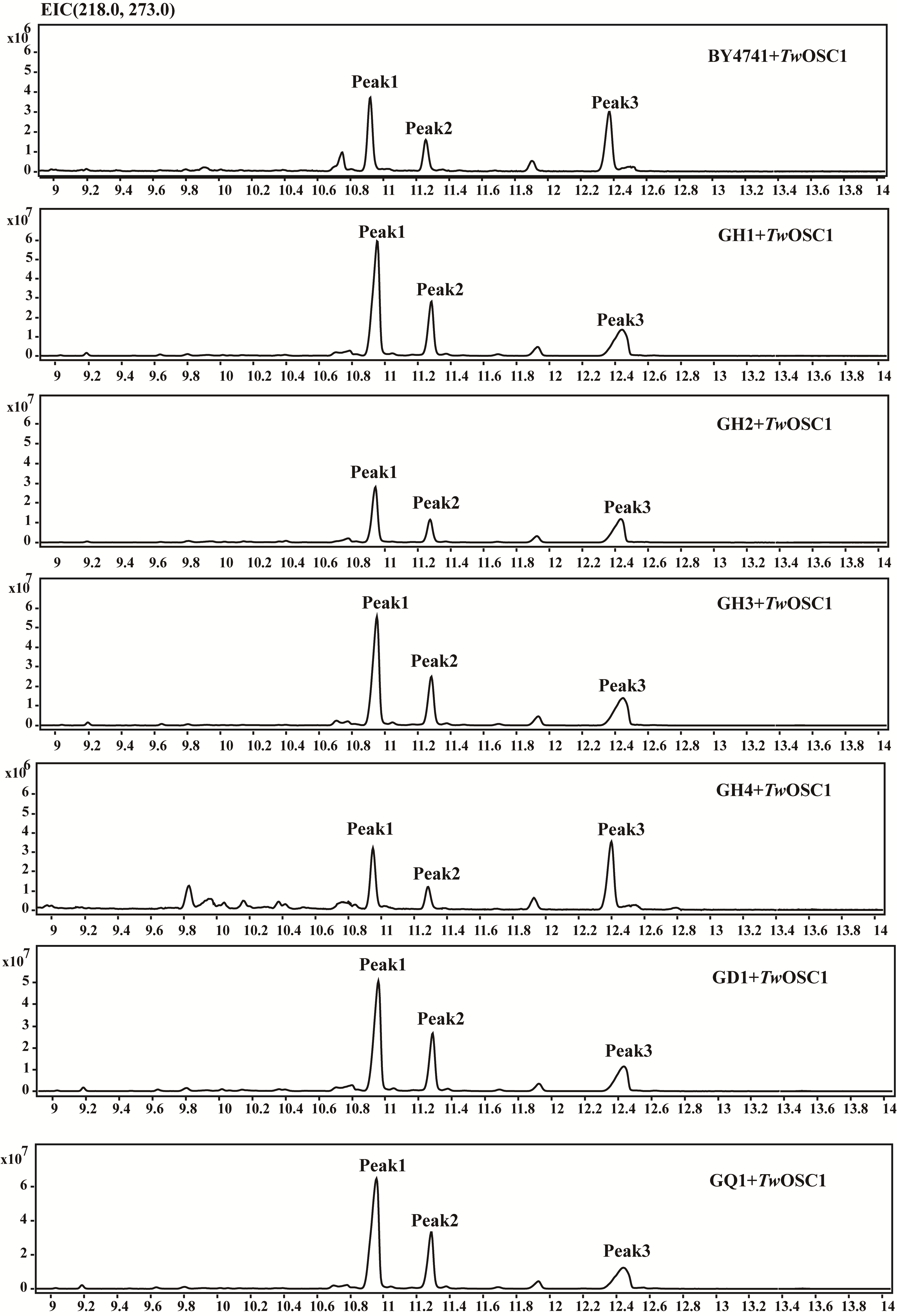


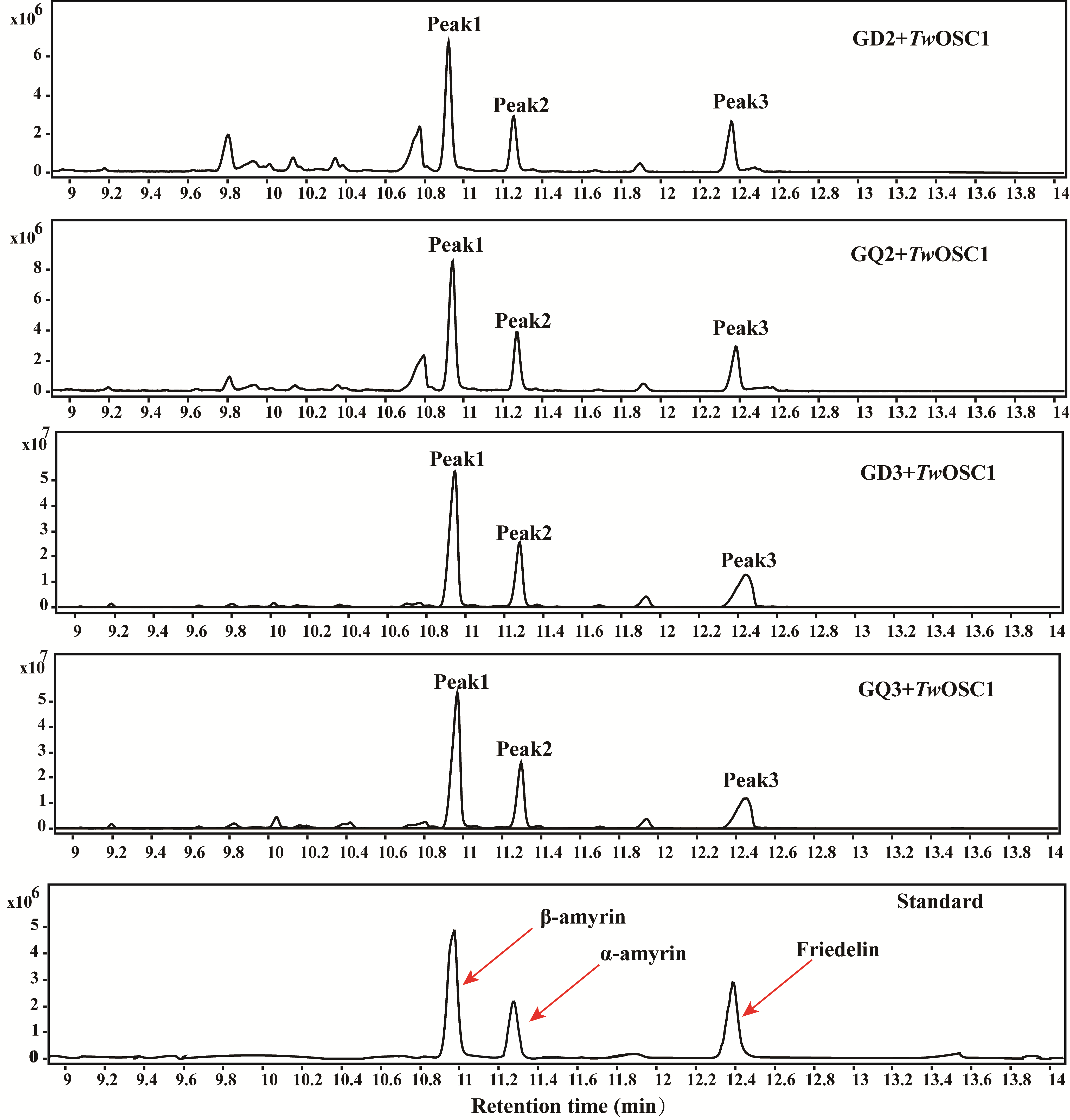


# Supplementary Figure S8. GC-MS analysis of *Tw*OSC1^T502E^ expressed in different recombinant strains. BY4741+*Tw*OSC1: Yeast strain BY4741 harbouring the *Tw*OSC1^T502E^. GH1+*Tw*OSC1: Yeast strain GH1 harbouring the *Tw*OSC1^T502E^. GH2+*Tw*OSC1: Yeast strain GH2 harbouring the *Tw*OSC1^T502E^. GH3+*Tw*OSC1: Yeast strain GH3 harbouring the *Tw*OSC1^T502E^. GH4+*Tw*OSC1: Yeast strain GH4 harbouring the *Tw*OSC1^T502E^. GD1+*Tw*OSC1: Yeast strain GD1 harbouring the *Tw*OSC1^T502E^ GQ1+*Tw*OSC1: Yeast strain GQ1 harbouring *Tw*OSC1^T502E^. GD2+*Tw*OSC1: Yeast strain GD2 harbouring the *Tw*OSC1^T502E^. GQ2+*Tw*OSC1: Yeast strain GQ2 harbouring *Tw*OSC1^T502E^. GD3+*Tw*OSC1: Yeast strain GD3 harbouring the *Tw*OSC1^T502E^. GQ3+*Tw*OSC1: Yeast strain GQ3 harbouring the *Tw*OSC1^T502E^.


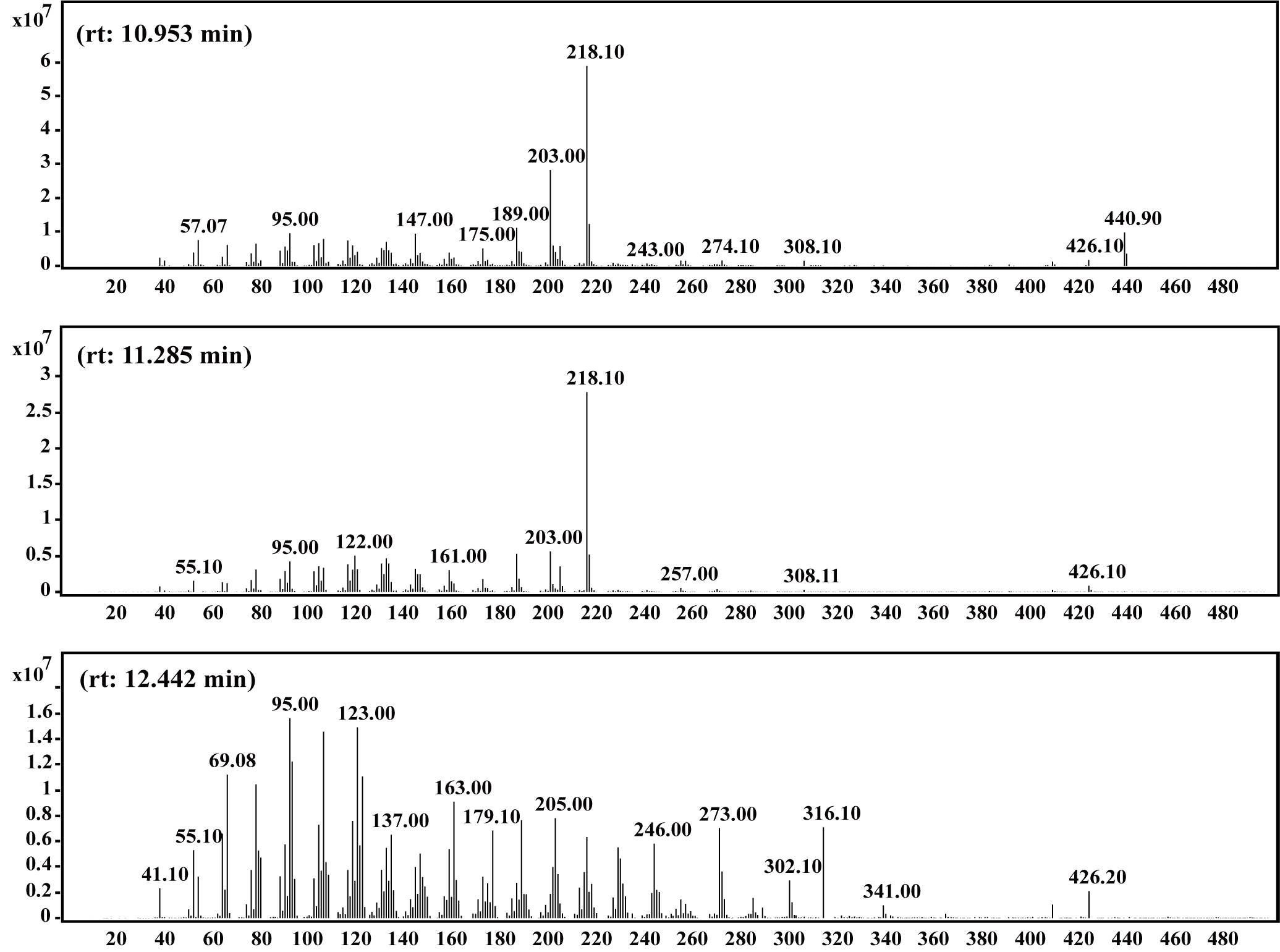


# Supplementary Figure S9. MS spectrum of extracts of *Tw*OSC1 ^T502E^ expressed in Recombinant yeast GH1 (The corresponding from top to bottom is peak1, peak2, peak 3).


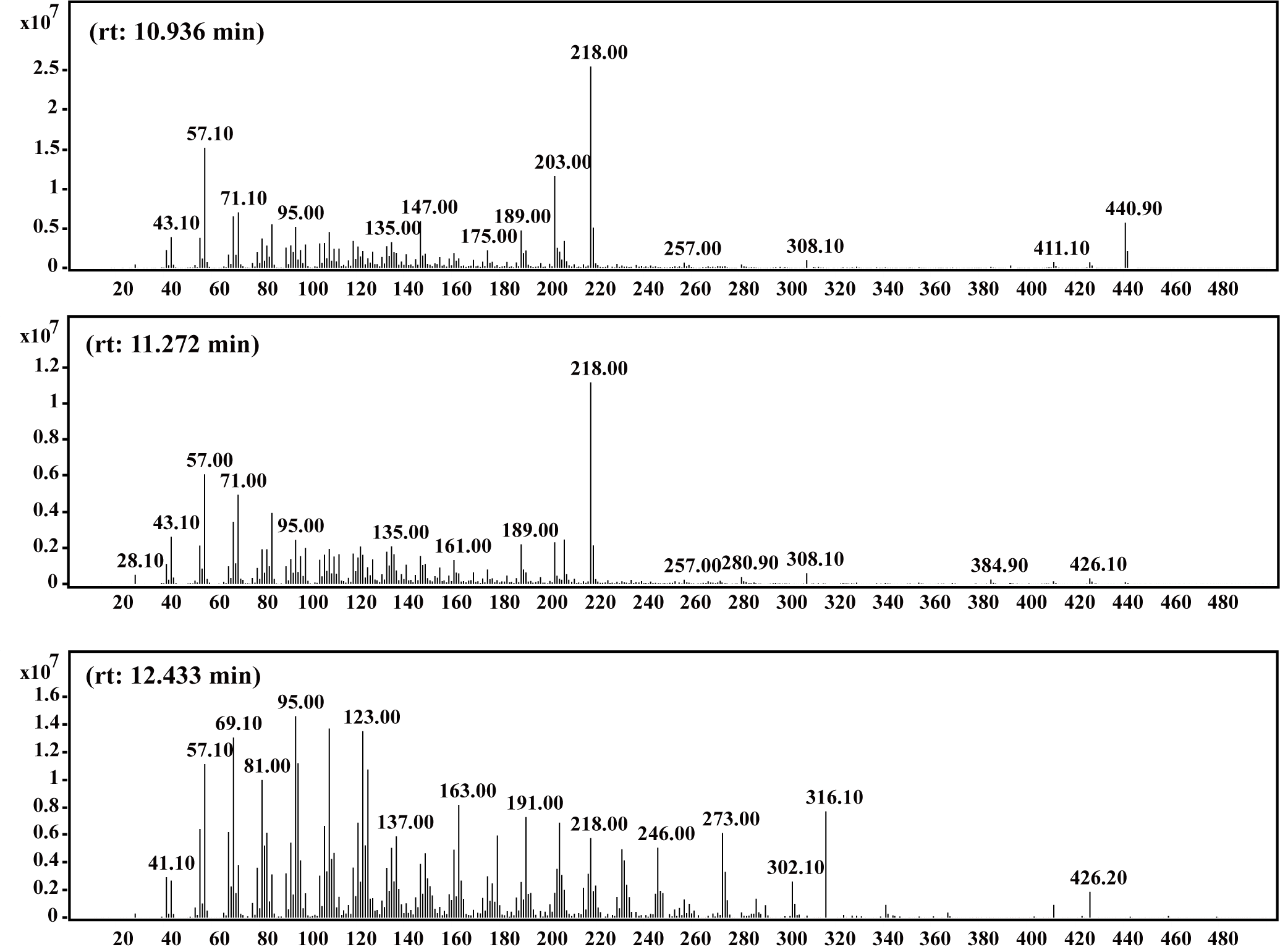


# Supplementary Figure S10. MS spectrum of extracts of *Tw*OSC1 ^T502E^ expressed in Recombinant yeast GH2 (The corresponding from top to bottom is peak1, peak2, peak 3).


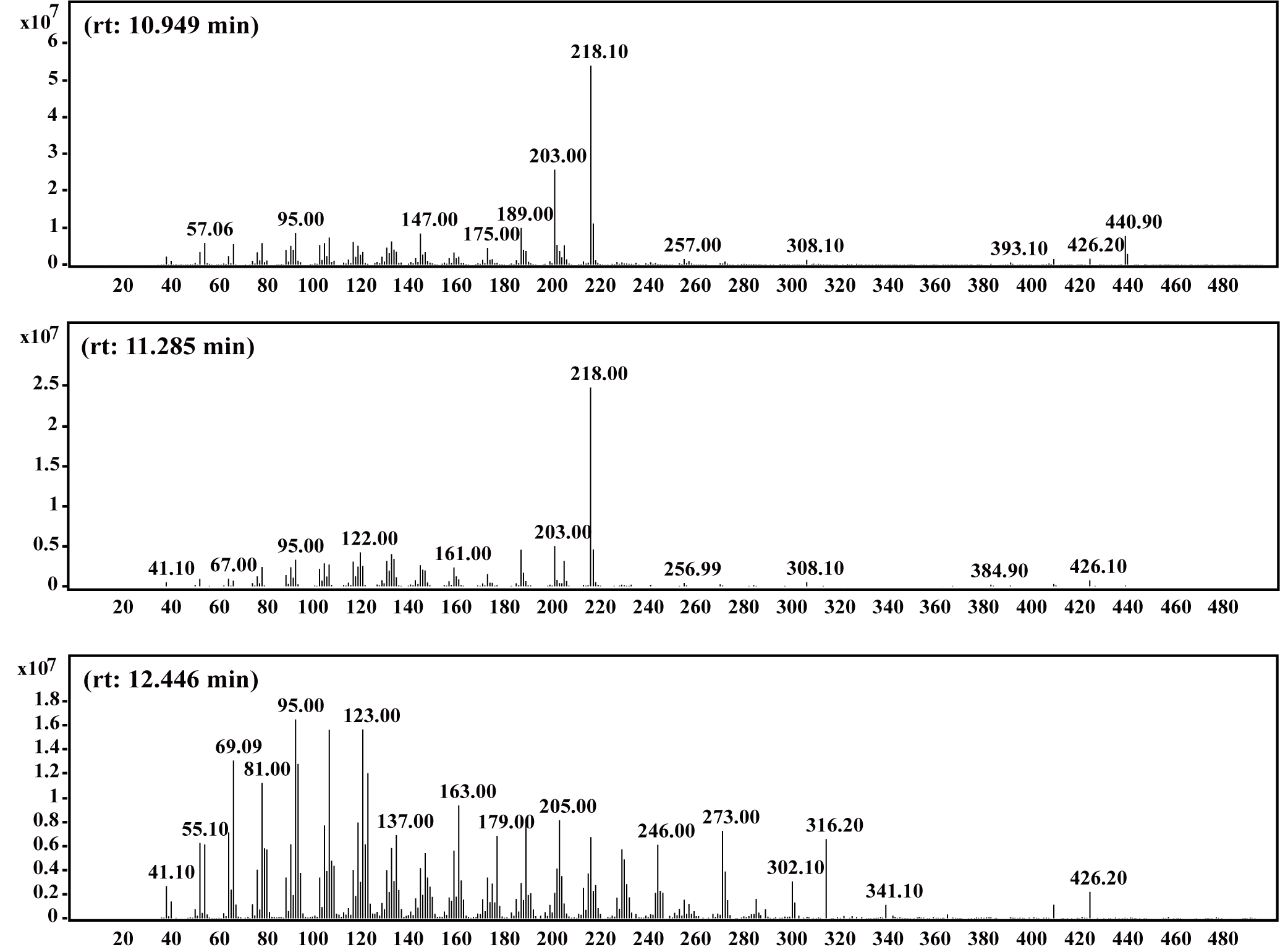


# Supplementary Figure S11. MS spectrum of extracts of *Tw*OSC1 ^T502E^ expressed in Recombinant yeast GH3 (The corresponding from top to bottom is peak1, peak2, peak 3).

**
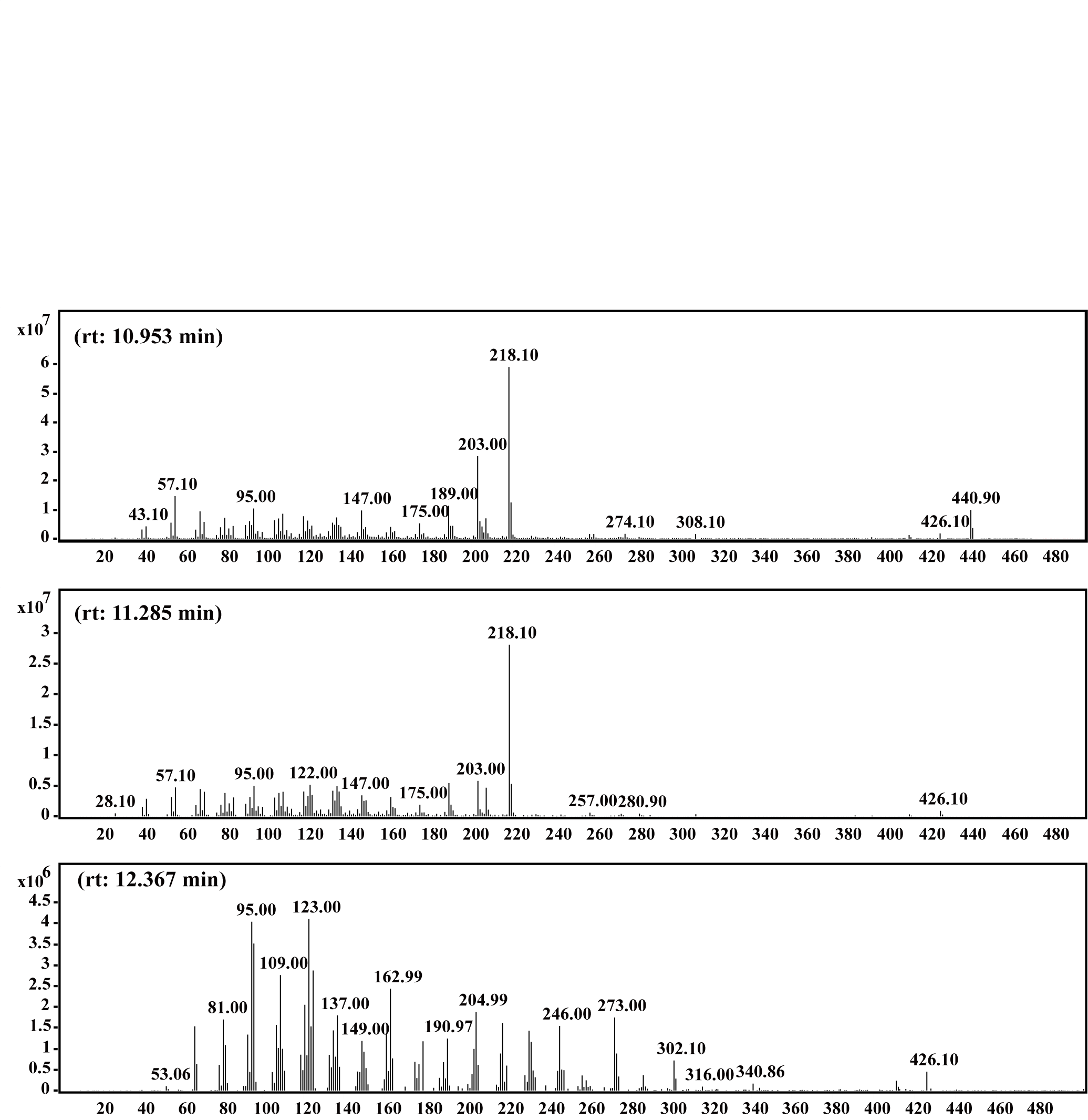
**

# Supplementary Figure S12. MS spectrum of extracts of *Tw*OSC1 ^T502E^ expressed in Recombinant yeast GH4 (The corresponding from top to bottom is peak1, peak2, peak 3).


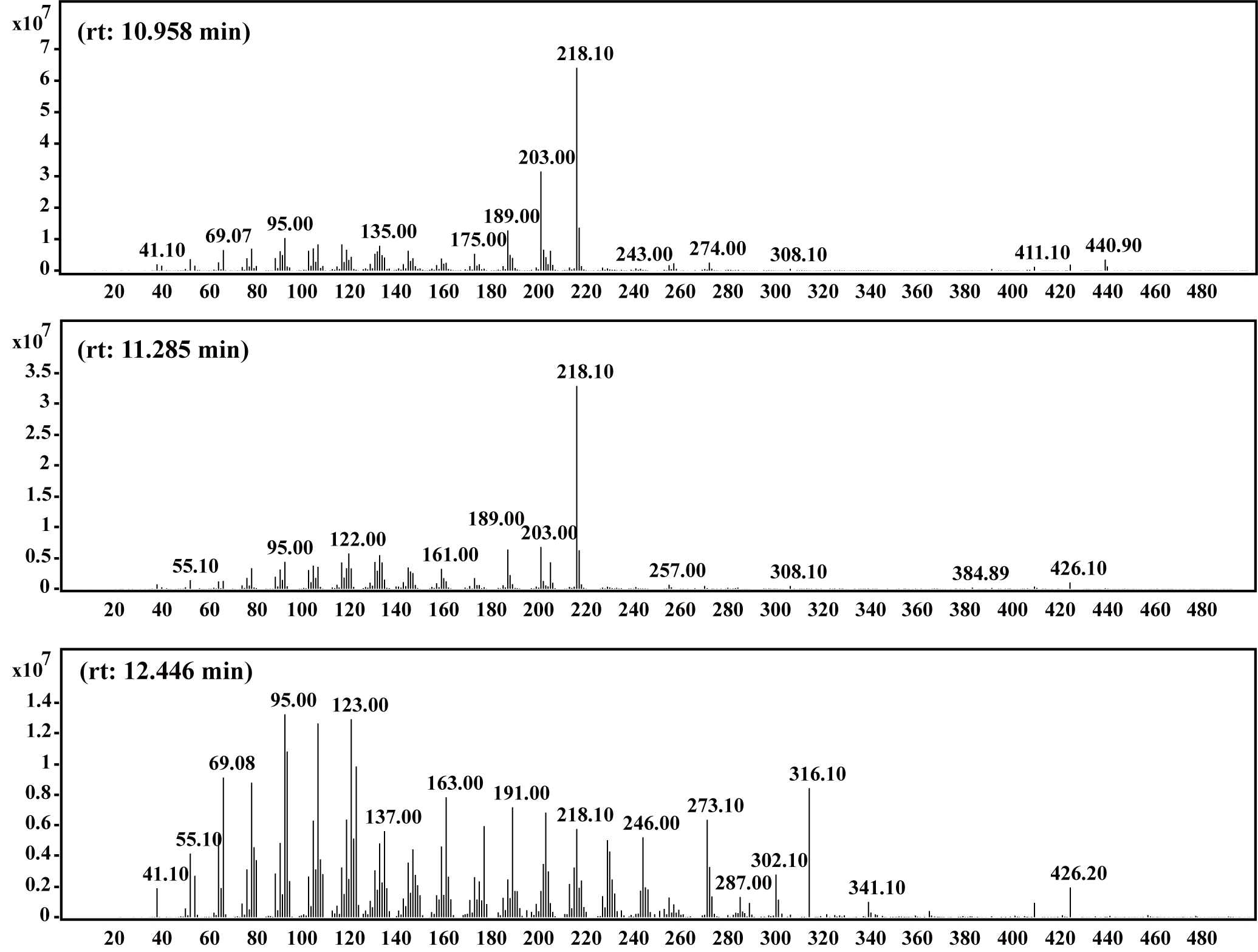


# Supplementary Figure S13. MS spectrum of extracts of *Tw*OSC1 ^T502E^ expressed in Recombinant yeast GD1 (The corresponding from top to bottom is peak1, peak2, peak 3).

**
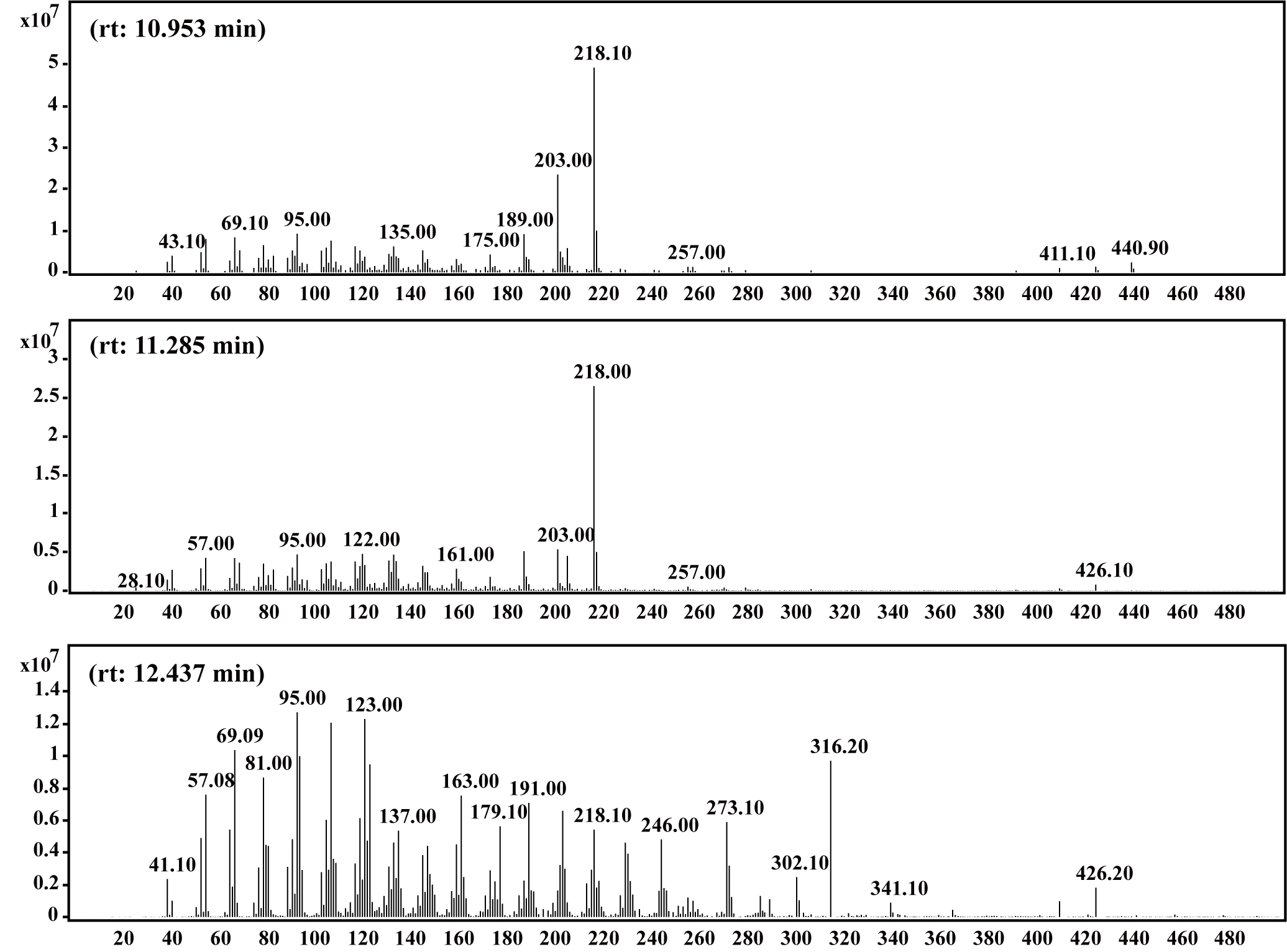
**

# Supplementary Figure S14. MS spectrum of extracts of *Tw*OSC1 ^T502E^ expressed in Recombinant yeast GQ1 (The corresponding from top to bottom is peak1, peak2, peak 3).

**
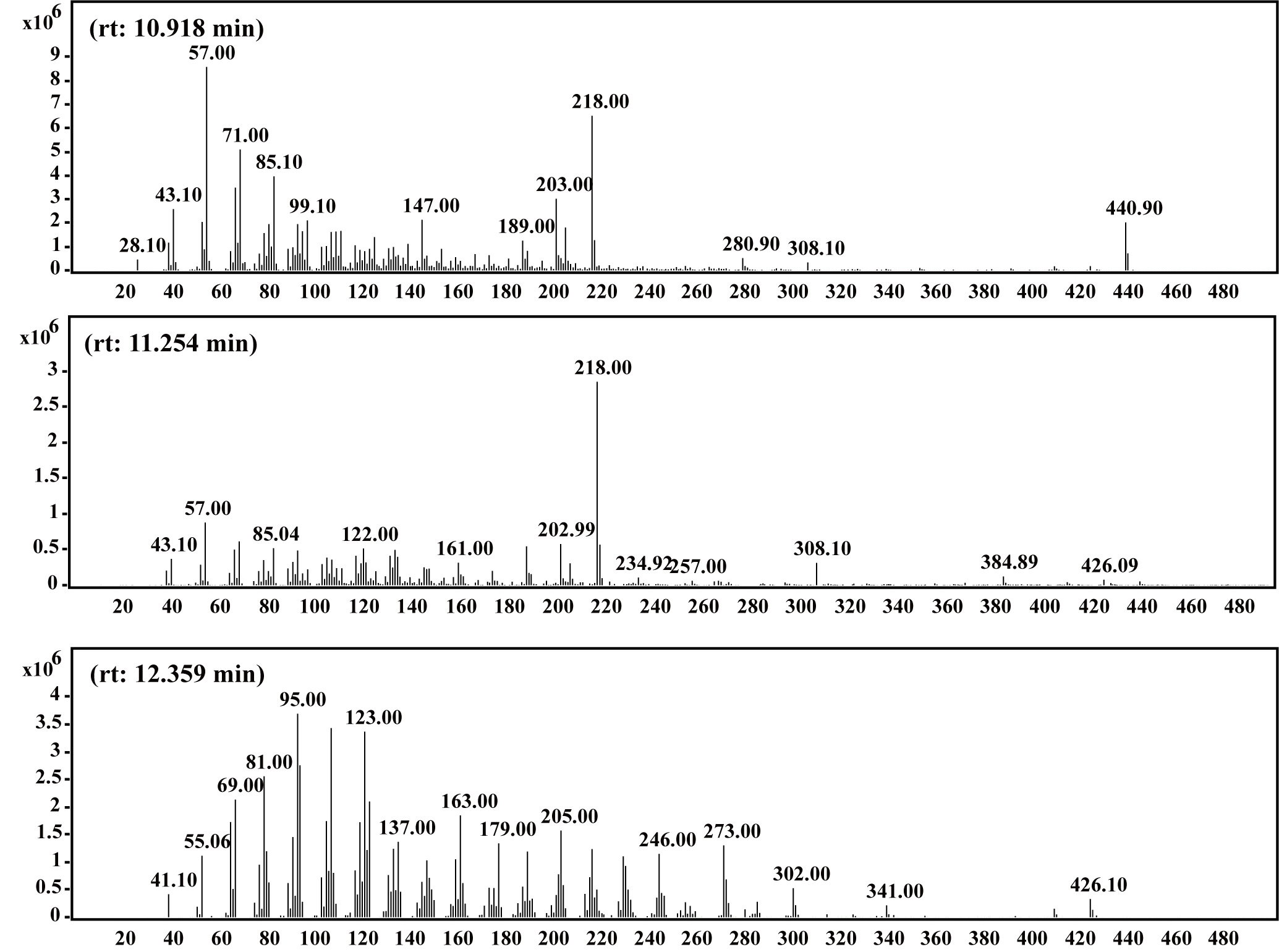
**

# Supplementary Figure S15. MS spectrum of extracts of *Tw*OSC1 ^T502E^ expressed in Recombinant yeast GD2 (The corresponding from top to bottom is peak1, peak2, peak 3).


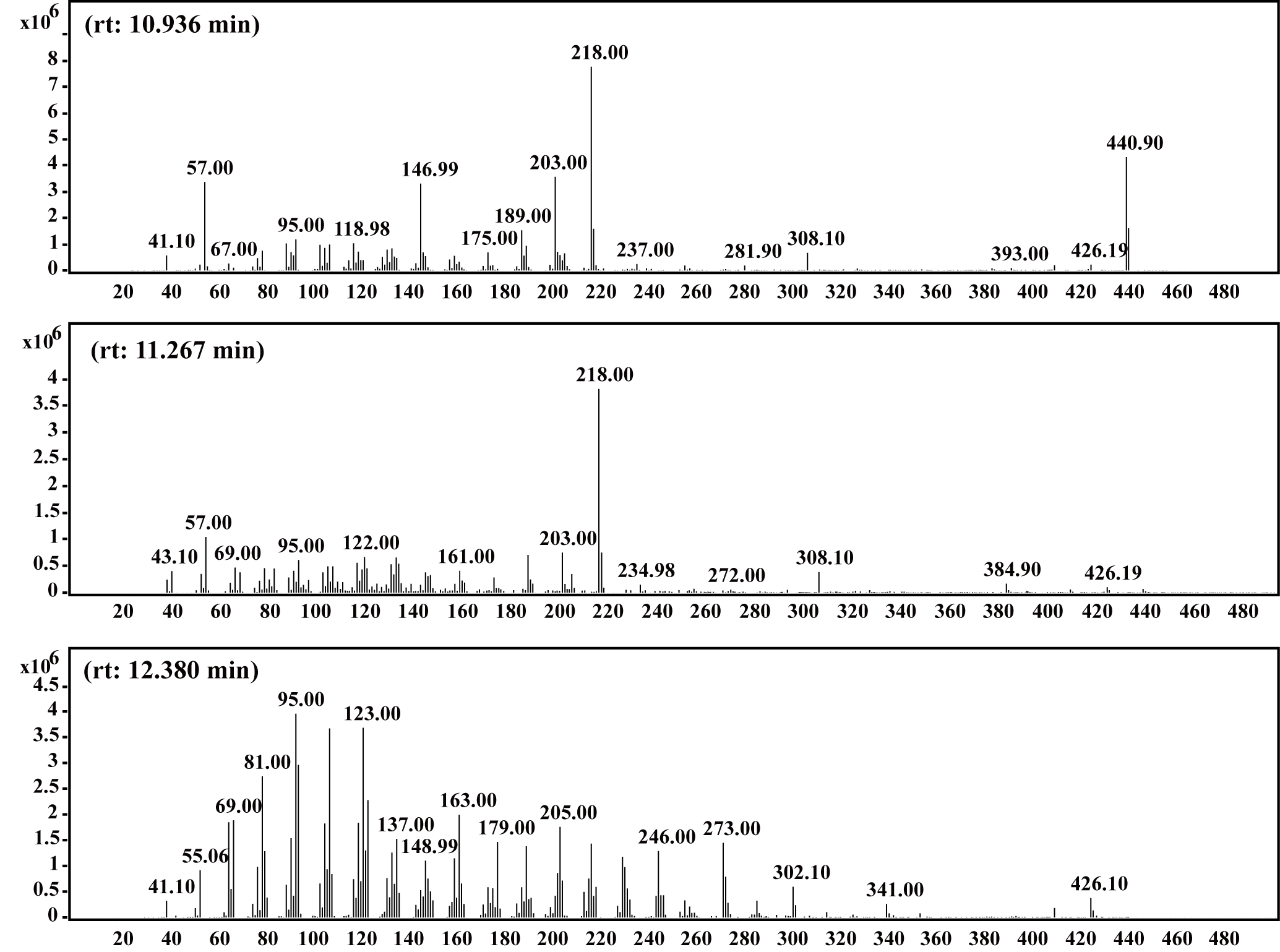


# Supplementary Figure S16. MS spectrum of extracts of *Tw*OSC1 ^T502E^ expressed in Recombinant yeast GQ2 (The corresponding from top to bottom is peak1, peak2, peak 3).


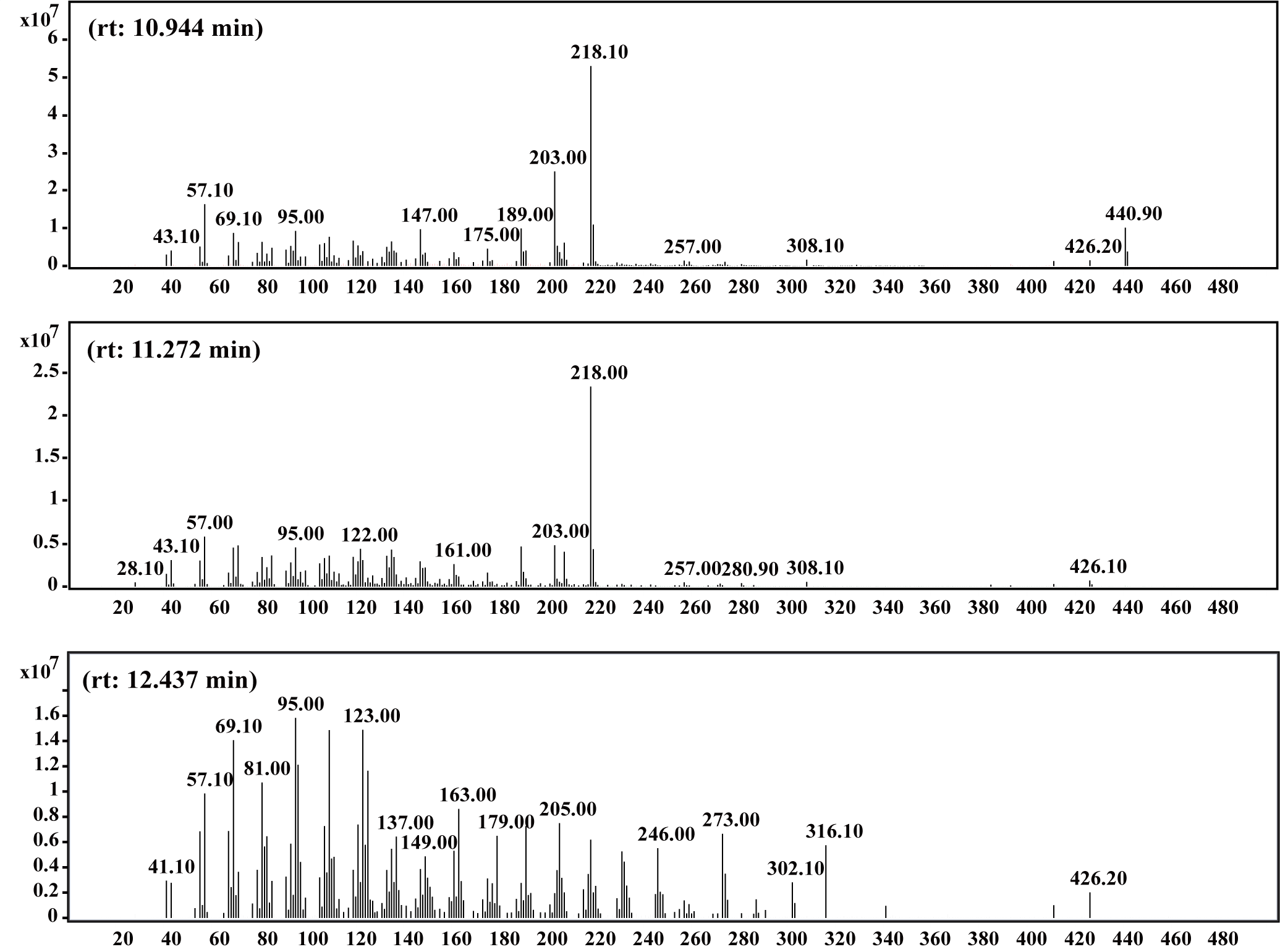


# Supplementary Figure S17. MS spectrum of extracts of *Tw*OSC1 ^T502E^ expressed in Recombinant yeast GD3 (The corresponding from top to bottom is peak1, peak2, peak 3).

**
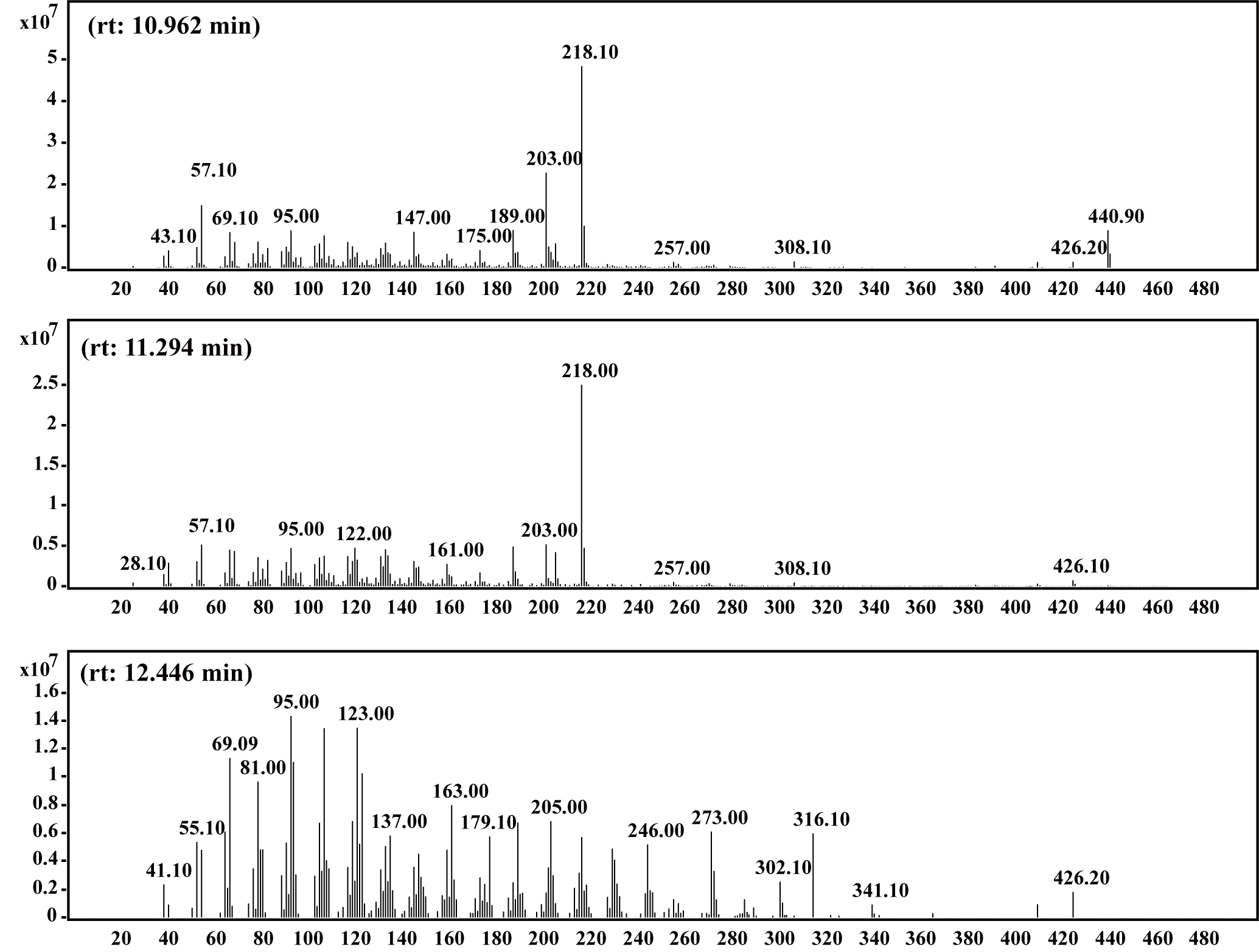
**

# Supplementary Figure S18. MS spectrum of extracts of *Tw*OSC1 ^T502E^ expressed in Recombinant yeast GQ3 (The corresponding from top to bottom is peak1, peak2, peak 3).

**
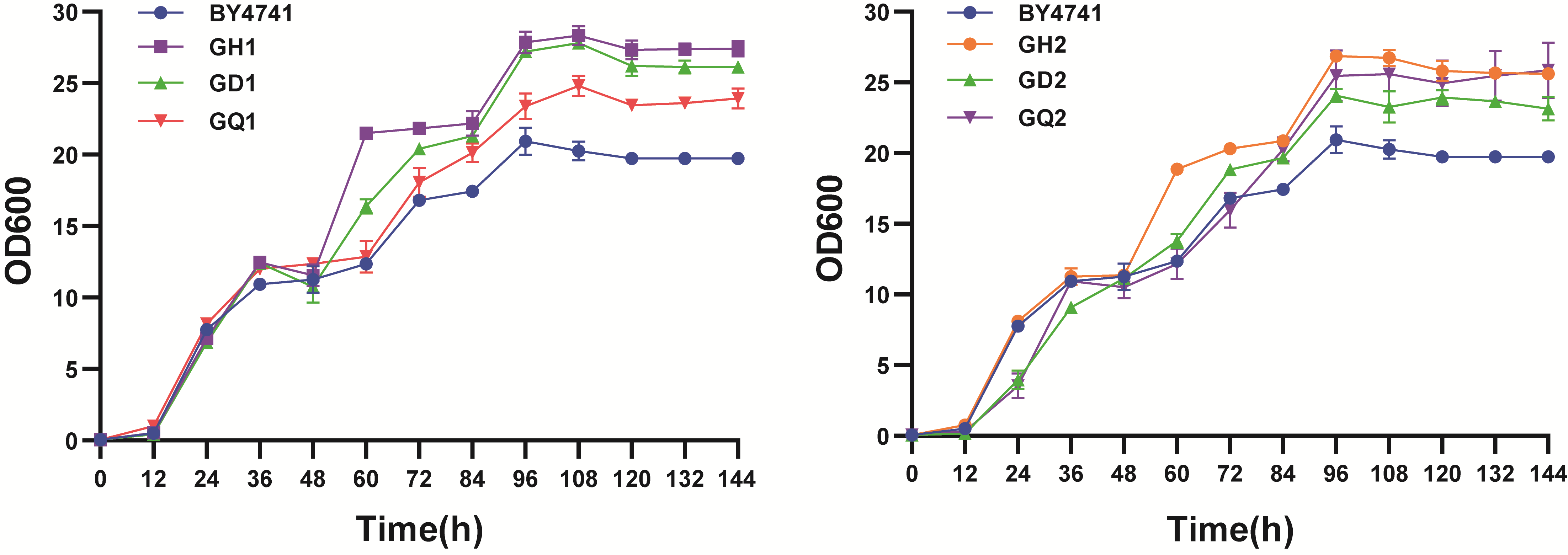
**

**
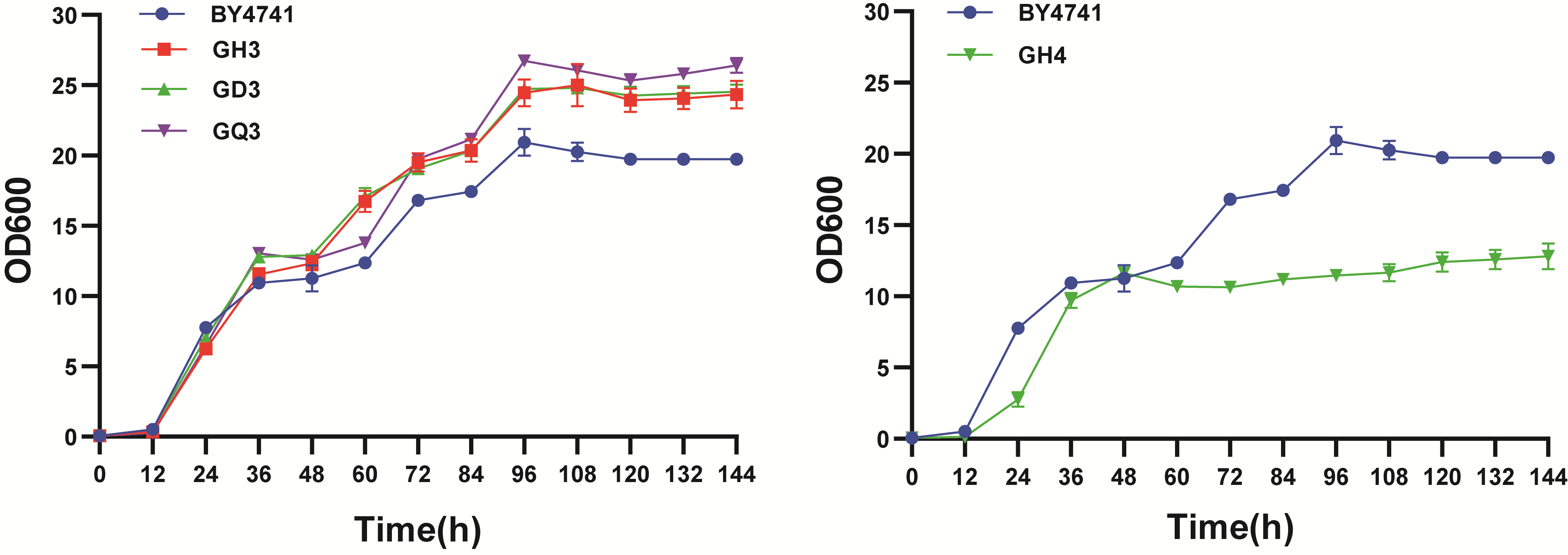
**

**
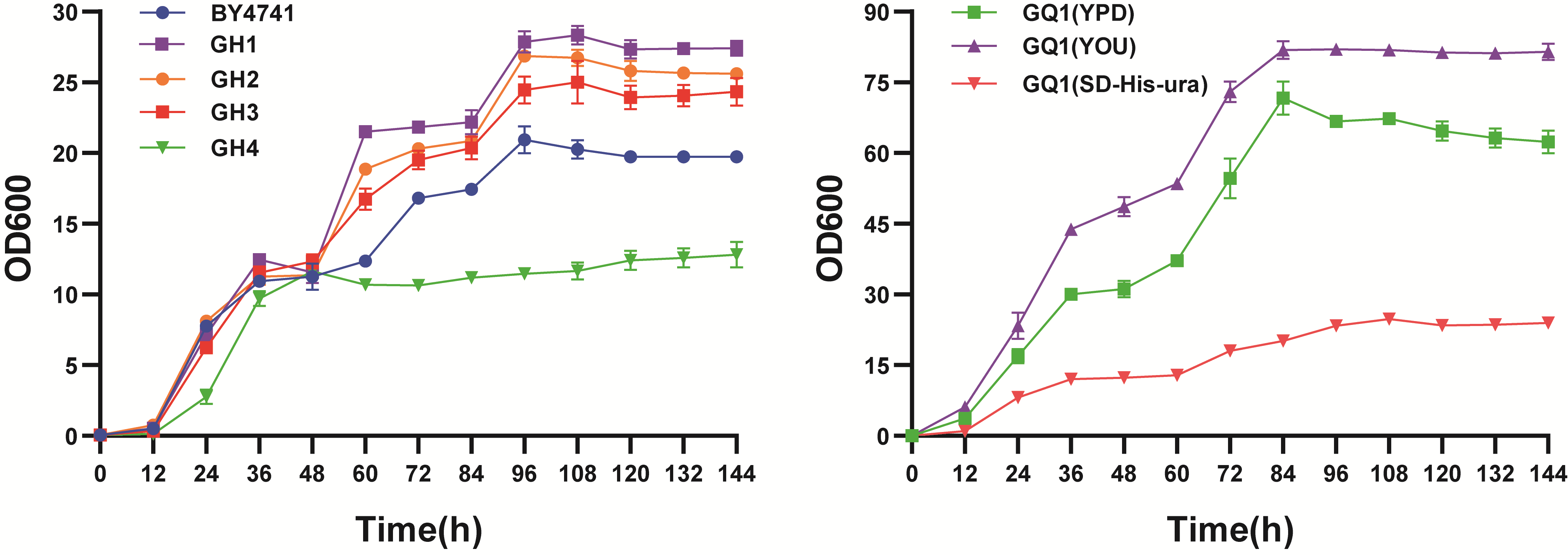
**

**Supplementary Figure S19. Comparison of growth curves of different strains.** The growth curves of different strains were compared, in which glucose was replaced with galactose at 48h. The data are the averages of 3 biological replicates with error bars representing standard deviations.

**Reference**

Li, S., Ding, W., Zhang, X., Jiang, H., Bi, C (2016). Development of a modularized two-step (M2S) chromosome integration technique for integration of multiple transcription units in *Saccharomyces cerevisiae*. Biotechnol Biofuels, 9: 232.

Zhou, Y.J., Gao, W., Rong, Q.X., Jin, G., Chu, H., Liu, W., Yang, W., Zhu, Z., Li, G., Zhu, G., Huang, L., Zhao, Z. K. (2012) Modular pathway engineering of diterpenoid synthases and the mevalonic acid pathway for miltiradiene production. J. Am. Chem. Soc. 134, 3234-3241.
